# Five new species of the leaf-beetle genus *Monolepta* Chevrolat (Coleoptera, Chrysomelidae, Galerucinae) from China

**DOI:** 10.3897/zookeys.1056.65335

**Published:** 2021-08-18

**Authors:** Qi-long Lei, Si-yuan Xu, Xing-ke Yang, Rui-E Nie

**Affiliations:** 1 Key Laboratory of Zoological Systematics and Evolution, Institute of Zoology, Chinese Academy of Sciences, 1 Beichen West Road, Chaoyang District, Beijing 100101, China Institute of Zoology, Chinese Academy of Sciences Beijing China; 2 University of Chinese Academy of Sciences, No. 19(A) Yuquan Road, Shijingshan District, Beijing, 100049, China University of Chinese Academy of Sciences Beijing China; 3 Guangdong Key Laboratory of Animal Conservation and Resource Utilization, Guangdong Public Laboratory of Wild Animal Conservation and Utilization, Guangdong Institute of Applied Biological Resources, Guangzhou 510260, China Guangdong Institute of Applied Biological Resources, Guangzhou China

**Keywords:** Coleoptera, Chrysomelidae, Galerucinae, Monoleptites, *
Monolepta
*, new species

## Abstract

In this study, five new species of the leaf-beetle genus *Monolepta* Chevrolat, 1836 (Coleoptera, Chrysomelidae, Galerucinae) are described from China: *M.albipunctata***sp. nov.**, *M.alticola***sp. nov.**, *M.bivittata***sp. nov.**, *M.mengsongensis***sp. nov.**, and *M.rubripennis***sp. nov.** A key and catalogue to the 68 Chinese species of *Monolepta* with the second and third antennomeres of equal length are given as well as photographs of the habitus and aedeagus of the new species and type habitus images of 37 known species.

## Introduction

With 708 species and six subspecies distributed worldwide (Nie et al. 2017), the leaf-beetle genus *Monolepta* is one of the largest genera in Galerucinae (Coleoptera, Chrysomelidae). There are 342 species distributed in the Oriental region, which is almost half the species in this genus. Several new Chinese species have been described since revision (Gressitt and Kimoto 1963) and 73 species have been recorded, 71 in the Oriental region and two in the Palaearctic region (Yang et al. 2015).

During sorting of specimens in the Institute of Zoology, Academy of Sciences, five new species were found and are described here. In addition, photographs of the habitus, external parts and aedeagus of the new species and habitus of known species (in Suppl. material 1) are also given together with a key to the Chinese species.

## Material and methods

The specimens were examined with an Olympus SZ61 microscope.

### Dissections

The abdomen was taken from the specimens, then transferred to a vial containing 5% NaOH solution and heated in boiled water around 5–7 minutes. The abdomen with aedeagus was washed in distilled water 3 or 4 times, transferred into a cavity slide using fine forceps and the aedeagus was separated by hooked minute-pin dissecting needles.

### Photographs

Habitus images were taken using a Canon 5DSR digital camera, equipped with a lens EF 75–300 mm f/4–5.6 linking a Nikon CFI Plan Apochromat Lambda 4× or 2× objective lens. Illumination was by flash, and each photo was taken by a macro slide system.

Aedeagus images were taken using a Nikon D610 digital camera, linking a Zeiss V microscope, with 5× and 10× objective lens. A cable shutter release was used to prevent the camera from shaking. The number of images taken was depending on the size of the aedeagus.

To get full depth of focus, all images were stacked with HELICON FOCUS 6 (http://www.heliconsoft.com/heliconsoft-products/helicon-focus/) and the resulting output, edited with Adobe Photoshop CC (https://www.photoshop.com/).

### Labels

The label data is translated into English from the original Chinese.

### Type depository

Type specimens of the five new species are deposited in the Institute of Zoology, Chinese Academy of Sciences, Beijing, China (**IZAS**).

#### Biology

The life cycle of most species of *Monolepta* is little known, but in China the life cycle of *M.signata* (Olivier, 1808) (= *M.hieroglyphica* (Motschulsky, 1858)) has been recorded in detail. It has one generation each year and overwinters as eggs, hatching in May. Its larvae live underground for about a month, feeding on grass roots. The mature larvae pupate in the soil after 7–10 days of emergence. Adults normally appear in July and survive until October (Research Group of Leaf Beetle 1979). Some species of this genus are important agriculture pests, for example *M.signata*, which is a widely distributed pest in Asia and causes serious damage to plants (such as *Arachishypogaea*, *Gossypium* sp., *Pyracanthacrenulate*, *Rubus* sp., *Salix* sp., *Viburnum* sp., *Zeamays*) in China, Nepal, and Bangladesh (Neupane et al. 2006; Zhang 2012), while *M.australis* Jacoby, 1882 is harmful to peanut crops in Queensland, Australia (Turner 1980).

## Taxonomy

### Genus Monolepta

Taxon classificationAnimaliaColeopteraChrysomelidae

Chevrolat

4342EE2D-DDC2-5FA1-92C3-43216C78211F

Monolepta Chevrolat 1836: 383. Type species: Criocerisbioculata Fabricius, 1781, by subsequent designation (Chevrolat 1845: 5).
Damais Jacoby 1903: 118. Type species: Damaishumeralis Jacoby, 1903, by monotypy. Synonymized by Maulik (1936: 373).
Aemulaphthona Scherer 1969: 89. Type species: Aemulaphthonaochracea (Weise, 1922), by monotypy. Synonymized by Konstantinov (2002: 210).
Chimporia Laboissière 1931: 413. Type species: Chimporiamonardi Laboissière, 1931, by monotypy. Synonymized by Wagner (2007: 84).

#### Distribution.

Palaearctic, Oriental, Australian, Afrotropical, Neotropical region.

#### Diagnosis.

Body length: 1.9–9.5 mm. Antennae longer than half or even equal to the body, segments 2 and 3 almost equal in length, segment 4 equal to or longer than sum of segments 2 and 3. Width of pronotum longer than length; anterior margin slightly depressed, basal margin protruding and lateral margins slightly protruding; basal margin and lateral margins with frame; anterior and posterior angle thickened, each angle with a seta-pore; disc convex, generally depressed on both sides. Scutellum triangular, smooth, and impunctate. Elytra broader than pronotum, humeral angle obvious; epipleuron broad before basal 1/3, then strongly narrowed and disappearing at beginning of apex. Anterior coxal cavities open or closed, each tibia with a spine in apex, spine of hind tibiae longest, 1^st^ segment of hind tarsi longer than remaining segments combined; claws appendiculate. Last sternite of male with trilobate concavities, female normal, without any concavities (Gressitt and Kimoto 1963).

#### Remarks.

Since its description, several genera have been synonymized with *Monolepta*. Of these, Maulik (1936) synonymized *Damais* Jacoby, 1903 based on the length of the 1^st^ segment of the hind tarsi which is longer than the remaining combined segments in *D.humeralis* Jacoby, 1903, the type species. Konstantinov (2002) synonymized *Aemulaphthona* Scherer, 1969, originally placed in Alticini, based on several characters of the type species *Aemulaphthonaochracea* (Weise, 1922), such as the flat head in lateral view, absence of a supraorbital sulcus, and metafemur without a metafemoral spring. Wagner (2007) synonymized *Chimporia*Laboissière 1931 based on the similarity of the aedeagus. Also, based on characters of the anterior coxae and the second antennomere, and morphology of aedeagus, many new genera were described for species previously included in *Monolepta*, such as *Afromaculepta* Wagner, 2000, *Afromegalepta* Wagner, 2001, *Afrocandezea* Wagner, 2002, *Afronaumannia* Wagner, 2005, *Monoleptoides* Wagner, 2011, *Neobarombiella* Wagner, 2012, *Orthoneolepta* Hazmi & Wagner, 2013, *Paraneolepta* Hazmi & Wagner, 2013, *Bicolorizea* Wagner, 2015, and *Doeberllepta* Wagner, 2017.

The ratio of antennomeres 2 and 3 is of great importance for the identification in *Monolepta* and related genera. The length of 2 to 3 in the type species, *M.bioculata*, is 0.83–1.00 (Wagner 2007). Sometimes antennomere 2 is slightly shorter than 3, as in *M.jeanneli* (0.78–0.87), or on the contrary, antennomere 2 is slightly longer than 3, as in *M.usambarica* (1.00–1.20; Wagner 2000). In general, the ratio of antennomeres 2 and 3 is 0.80–1.20.

There are also some similar genera in the Oriental region. In *Arcastes*, the lack of pronotal depressions and the significantly enlarged antennomeres 3–8 distinguishes it from other genera, as does the ratio of antennomeres 2 and 3, which is 0.5–0.57; thus, it is easily recognized from *Monolepta* (Hazmi and Wagner 2010a). *Rubrarcastes* has the similarly enlarged antennomeres of *Arcastes*, but the ratio of antennomeres 2 and 3 is 0.43–0.57 (Hazmi and Wagner 2010b). In the Oriental region, the relatively large body and the transverse depression on the pronotum distinguish *Paraneolepta*; antennomeres 4–6 are significantly widened in *Orthoneolepta*, which is different from that of *Monolepta*. In *Ochralea* Clark, 1865 the relatively large body (7.75–14.40 mm) and the deeply incised median lobe of aedeagus are characteristic (Hazmi and Wagner 2010c). The ratio of antennomeres of *Neolepta* is 0.75–0.80, *Paraneolepta* is 0.75–0.86, and *Orthoneolepta* 0.60–1.00. *Neolepta* is usually with widened median antennomeres. However, these three similar genera have a tansverse depression on pronotum, which is not present in *Monolepta*.

Eleven similar genera are distributed in China. In *Atrachya* Dejean, 1837, antennomere 3 is much longer than 2, and the tectum is deeply incised and with strong apical hooks (Lee 2020). In *Sermyloides* Jacoby, 1884, there is a strong frontal depression in males and a usually modified antennomere 3. In *Ochralea* Clark, 1865 antennomeres 2 and 3 are almost equal in length. In *Shaira* Maulik, 1936, the elytra is very short, and so this genus can be easily distinguished. In *Pseudosepharia* Laboissière, 1936, the epipleuron is very broad and 1/3 times as wide as the elytron. In *Paleosepharia* Laboissière, 1936, the epipleuron is gradually narrowed from its base to its apex, and there is sexual dimorphism (Lee 2018). In *Macrima* Baly, 1878, there is a frontal depression in males. *Trichosepharia* Laboissière, 1936 has the basal part of the median lobe incised and the tectum enlarged at its apex. In *Chinochya* Lee, 2020, tasomere 1 is swollen in males, and there are two types of endophallic spiculae. *Tsouchya* Lee, 2020, has antennomere 2 much shorter than 3, and there are two types of endophallic spiculae. In *Neochya* Lee, 2020 antonnomeres 2 and 3 are almost the same length, but the coxal cavities are widely open and there is only one pair of endophallic spiculae.

The species included in *Monolepta* generally have two types of antennae: either with segments 2 and 3 equal in length or with segment 3 longer than 2. Most species of the former group have a similar type of aedeagus; these include: *M.babai* Kimoto, 1996; *M.bicavipennis* Chen, 1942; *M.kwangtunga* Gressitt & Kimoto, 1963; *M.mordelloides* Chen, 1942; *M.parvezi* Aslam, 1968, and *M.subflavipennis* Kimoto, 1989. Since the redescription of the type species by Wagner (2007), “true” *Monolepta* can be distinguished by the similar lengths of antennomeres 2 and 3, the abruptly narrowed epipleuron after the basal 1/3, and the aedeagus type. Although, the closed anterior coxal cavities of *Monolepta* were the main character to identify the genus in the past, Wagner (1999, 2007) redescribed anterior coxal cavities of the type species and showed them to be open. So, these structures are rather variable, with some closed or almost closed and others completely open.

Although 73 species of *Monolepta* are known from China, little recent detailed work on the genus has so far been published, and some species with the second and third antennomeres of unequal length and different types of aedeagus may need to be transferred to other genera in the future, for example *M.yaosanica* Chen, 1942 and *M.postfasciata* Gressitt & Kimoto, 1963. The following key is restricted to those 68 species which have antennomeres 2 and 3 of equal length.

### Key to the species of Chinese *Monolepta*

Note: the key only includes species with the second and the third antennomeres approximately equal in length (see generic Remarks).

**Table:** 

1	Elytra with depressions	**2**
–	Elytra without depressions	**3**
2	Elytra yellow, with three transverse black bands (Suppl. material 1: Fig. S6)	***M* . *cavipennis* Baly, 1878**
–	Elytra orange red, with two kidney-shaped depressions, one before middle suture, another outside of middle suture (Suppl. material 1: Fig. S37)	***M.quadricavata* Chen, 1976**
3	Elytra entirely red	**4**
–	Elytra yellowish brown, reddish brown or black	**5**
4	Pronotum black, elytra red (Fig. 29)	***M.rubripennis* sp. nov.**
–	Pronotum orange red, elytra with a pale-yellow dot near apex (Suppl. material 1: Fig. S11)	***M.eunicia* Maulik, 1936**
5	Elytra black	**6**
–	Elytra yellowish brown, reddish brown or partially black	**16**
6	Head yellow (Suppl. material 1: Fig. S51)	***M.yaosanica* Chen, 1942**
–	Head black or partial black	**7**
7	Head partially black	**8**
–	Head black, body wide oval (Suppl. material 1: Fig. S43)	***M.semenovi* Ogloblin, 1936**
8	Head partially black	**9**
–	Head yellow, yellowish brown or reddish brown	**12**
9	Pronotum dark brown	**10**
–	Pronotum yellowish brown or reddish brown	**11**
10	Abdomen yellowish brown	***M.asahinai* Chûjô, 1962**
–	Abdomen black (Suppl. material 1: Fig. S14)	***M.horni* Chûjô, 1938**
11	Head black, frontal area dark yellowish brown (Suppl. material 1: Fig. S9)	***M.epistomalis* Laboissière, 1934**
–	Head yellowish brown (Fig. 22)	***M.alticola* sp. nov.**
12	Abdomen yellowish brown	**13**
–	Abdomen black or dark brown	**14**
13	Scutellum yellowish brown (Suppl. material 1: Fig. S41)	***M.schereri* Gressitt & Kimoto, 1963**
–	Scutellum black	***M.longicornis* (Jacoby, 1890)**
14	Legs black (Suppl. material 1: Fig. S10)	***M.erythrocephala* (Baly, 1878)**
–	Legs reddish brown	**15**
15	Antennae yellowish brown, segments 9–11 darker (Suppl. material 1: Fig. S32)	***M.ovatula* Chen, 1942**
–	Antennae black, basal 4–5 segments yellowish brown	***M.chinkinyui* Kimoto, 1996**
16	Elytra yellow or reddish brown, without any bands	**17**
–	Elytra with yellow or with black bands	**39**
17	Head black or partially black	**18**
–	Head not black	**20**
18	Occiput reddish brown (Suppl. material 1: Fig. S27)	***M.meridionalis* Gressitt & Kimoto, 1963**
–	Occiput black or head entirely black	**19**
19	Pronotum yellow; punctures of head stronger than that of elytra (Suppl. material 1: Fig. S50)	***M.xanthodera* Chen, 1942**
–	Pronotum red; punctures of head finer than that of elytra (Suppl. material 1: Fig. S5)	***M.capitata* Chen, 1942**
20	Abdomen black	**21**
–	Abdomen not black	**22**
21	Apex of elytra truncate (Suppl. material 1: Fig. S48)	***M.subrubra* Chen, 1942**
–	Apex of elytra rounded	***M.mandibularis* Chûjô, 1962**
22	Body usually small, less than 8 mm	**23**
–	Body very large, 9.5 mm	***M.severini* (Jacoby, 1896)**
23	Body length less than 2.5 mm	**24**
–	Body length more than 3.0 mm	**26**
24	Elytral punctures arranged in irregular longitudinal rows (Suppl. material 1: Fig. S29)	***M.minutissima* Chen, 1942**
–	Elytral punctures not arranged in rows	**25**
25	Pronotum punctures larger than elytral ones; punctures of elytra not combined (Suppl. material 1: Fig. S28)	***M.minor* Chûjô, 1938**
–	Pronotum punctures finer than elytral ones; some punctures of elytra combined (Suppl. material 1: Fig. S4)	***M.brittoni* Gressitt & Kimoto, 1963**
26	Antennae black, yellowish, or reddish brown	**27**
–	Antennae yellowish or reddish brown, except basal 3 segments black	***M.indochinensis* Medvedev, 1999**
27	Antennae black	**28**
–	Antennae yellowish brown or reddish brown	**31**
28	General color reddish brown	**29**
–	General color yellowish brown	**30**
29	Elytra with strong punctures; abdomen without long hairs	***M.annamita* Laboissière, 1935**
–	Elytra with fine punctures; abdomen with long hairs	***M.meihuai* Lee, Tian & Staines, 2010**
30	Apex of aedeagus constricted dorsally (Suppl. material 1: Fig. S38)	***M.rufofulva* Chûjô, 1938**
–	Apex of aedeagus expanded dorsally, constricted near apex	***M.nakanei* Kimoto, 1969**
31	Antennomere 4 longer than or equal to the sum of 2 and 3	**32**
–	Antennomere 4 shorter than the sum of 2 and 3 (Suppl. material 1: Fig. S18)	***M.lauta* Gressitt & Kimoto, 1963**
32	Pronotum yellowish brown, lateral margins black (Suppl. material 1: Fig. S31)	***M.ongi* Lee & Staines, 2010**
–	Pronotum yellowish brown, without any color margin	**33**
33	Antennomere 3 as long as 2	**34**
–	Antennomere 3 1.3 times as long as 2 (Suppl. material 1: Fig. S33)	***M.pallidula* (Baly, 1874)**
34	Body length less than 3.5 mm	**35**
–	Body length more than 5.5 mm (Suppl. material 1: Fig. S7)	***M.cheni* Beenen, 2008**
35	Ventral side of mesothorax yellow or brown	**36**
–	Ventral side of mesothorax black	***M.hongkongensis* Kimoto, 1967**
36	Ventral side of mesothorax yellow	**37**
–	Ventral side of mesothorax brown (Suppl. material 1: Fig. S3)	***M.arundinariae* Gressitt & Kimoto, 1963**
37	Space between elytral punctures equals to or larger than diameter of punctures	**38**
–	Space between elytral punctures less than diameter of punctures (Suppl. material 1: Fig. S34)	***M.palliparva* Gressitt & Kimoto, 1963**
38	Space between punctures equals to diameter of punctures (Suppl. material 1: Figs S15, S16)	***M.hupehensis* Gressitt & Kimoto, 1963**
–	Space between punctures 3 times as diameter of punctures (Suppl. material 1: Fig. S1)	***M.aglaonemae* Gressitt & Kimoto, 1963**
39	The apical area of elytra mostly black	**40**
–	The apical area of elytra not black	**44**
40	Head partially black or not black	**41**
–	Head black	***M.bacboensis* Medvdev, 2012**
41	Head partially black	**42**
–	Head not black	**43**
42	Vertex black, basal 2/3 of elytra reddish brown	***M.yama* Gressitt & Kimoto, 1965**
–	Frontal area black, basal 2/3 of elytra yellowish brown (Suppl. material 1: Fig. S42)	***M.selmani* Gressitt & Kimoto, 1963**
43	Ventral surface of mesothorax and metathorax black, basal 1/2 of elytra reddish brown, apical 1/2 black (Suppl. material 1: Fig. S39)	***M.sasajii* Kimoto, 1969**
–	Ventral surface of mesothorax and metathorax yellowish brown, basal 3/5 of elytra yellowish brown, apical 1/2 dark brown (Suppl. material 1: Fig. S47)	***M.subapicalis* Gressitt & Kimoto, 1963**
44	Elytra with colorful border	**45**
–	Elytra with black markings	**51**
45	Ventral side of mesothorax yellowish brown	**46**
–	Ventral side of mesothorax black (Suppl. material 1: Fig. S49)	***M.wilcoxi* Gressitt & Kimoto, 1965**
46	Lateral margin of pronotum has the same color as pronotum	**47**
–	Lateral margin of pronotum black	***M.takizawai* Kimoto, 1996**
47	Antennae reching more than 2/3 of elytra	48
–	Antennae reaching middle of elytra	***M.weigeli* Medvedev, 2012**
48	Antennae almost as long as body	**49**
–	Antennae not reaching apical 2/3 of elytra	**50**
49	Elytra yellowish brown, 4/5 lateral margin of elytra black (Suppl. material 1: Fig. S17)	***M.kuroheri* Kimoto, 1966**
–	Elytra yellowish brown, 2/5 lateral margin of elytra black (Suppl. material 1: Fig. S40)	***M.sauteri* Chûjô, 1935**
50	Ventral surface of mesothorax and metathorax black (Suppl. material 1: Fig. S2)	***M.alnivora* Chen, 1976**
–	Ventral surface of metathorax black, mesothorax yellow (Suppl. material 1: Fig. S13)	***M.gracilipes* Chûjô, 1938**
51	Elytra with black stripes or bands	**52**
–	Elytra with black or yellowish-brown spots	**58**
52	Pronotum reddish brown	**53**
–	Pronotum yellowish brown, each elytron with a semicircle spot in the middle, apex with a black parentheses-shaped marking (Suppl. material 1: Fig. S35)	***M.parenthetica* Gressitt & Kimoto, 1963**
53	Elytra black, with two pale spots in basal and apical area; or apex not black, with yellow spots in basal part (Suppl. material 1: Fig. S46)	***M.signata* (Olivier, 1808)**
–	Elytron without above characters	**54**
54	Pronotum reddish brown	**55**
–	Pronotum yellowish brown	**62**
55	Elytra without two black transverse bands	**56**
–	Elytra with two black transverse bands (Fig. 8)	***M.bivittata* sp. nov.**
56	Elytra with a black thin longitudinal band (Suppl. material 1: Fig. S44)	***M.sexlineata* Chûjô, 1938**
–	Elytra bands not longitudinal	**57**
57	Apex and base of elytra with a black band, middle yellow (Suppl. material 1: Fig. S30)	***M.occifluvis* Gressitt & Kimoto, 1963**
–	Basal 1/6 of elytra and apical 1/6 with black bands, middle with transverse brown and yellow bands (Suppl. material 1: Fig. S53)	***M.zonalis* Gressitt & Kimoto, 1963**
58	Elytra with a small spot near base, a slightly larger spot after middle (Suppl. material 1: Fig. S23, S24)	***M.longitarsoides* Chûjô, 1938**
–	Elytra with more than two black spots or with yellow or brown bands	**59**
59	Elytra with black spots between humeral angle and middle suture (Suppl. material 1: Fig. S45)	***M.shaowuensis* Gressitt & Kimoto, 1963**
–	Elytra with yellow or brown bands	**60**
60	Abdomen not black	**61**
–	Abdomen black; basal 2/3 of elytra black, with a yellow spot	***M.quadriguttata* (Motschulsky, 1860)**
61	Basal part of elytra red, turn black gradually to apex, with a rounded spot in middle (Suppl. material 1: Fig. S25)	***M.lunata* Gressitt & Kimoto, 1963**
–	Middle part of elytra with a “T” shape black stripes (Suppl. material 1: Fig. S36)	***M.postfasciata* Gressitt & Kimoto, 1963**
62	Abdomen black	***M.discalis* Gressitt & Kimoto, 1963**
–	Abdomen yellowish brown or reddish brown	**63**
63	Antennae as long as body (Fig. 15)	***M.mengsongensis* sp. nov.**
–	Antennae not reaching apical 2/3 of elytra	**64**
64	Antennae not reaching to half length of body	**65**
–	Antennae reaching more than half length of body	**66**
65	Tibiae and tarsi yellowish brown (Suppl. material 1: Fig. S26)	***M.maana* Gressitt & Kimoto, 1963**
–	Fore-legs yellowish brown, apex of tibiae and tarsi dark brown; coxae to femurs of meso and meta-legs yellow, tibiae and tarsi dark brown (Suppl. material 1: Figs S19, S20)	***M.leechi* Jacoby, 1890**
66	Elytra without any transverse bands	**67**
–	Elytra dark brown with a transverse yellow band (Suppl. material 1: Figs S21, S22)	***M.liui* Gressitt & Kimoto, 1963**
67	Elytra yellowish brown with wide dark brown frame (Suppl. material 1: Fig. S12)	***M.flavovittata* Chen, 1942**
–	Elytra black with a yellow dot in the middle (Suppl. material 1: Fig. S52)	***M.yunnanica* Gressitt & Kimoto, 1963**

### Description of new species

#### Monolepta albipunctata sp. nov.

Taxon classificationAnimaliaColeopteraChrysomelidae

7DDD5A1C-592F-586B-9EF4-60B3FD550F2A

http://zoobank.org/C2EB97A0-B103-4666-8451-1082316A27C3

Figs 1–10

##### Type material.

***Holotype***: China • ♂; Guangxi, Jinxiu, Luoxiang; 400 m; 14-V-1999; Xing-ke Yang leg. (IZAS). ***Paratypes***: China • 1♂; same data as holotype • 1♀; Guangxi, Jinxiu, Luoxiang; 450 m; 30-VI-2000; Jun Chen leg. • 2♀♀; Guangxi, Jinxiu, Luoxiang; 400 m; 15-V-1999; Da-jun Liu leg. (all IZAS).

##### Description.

Length: 5.5–6.6 mm, width 2.7–3.7 mm. Holotype: length 6.6 mm, width 3.4 mm.

Head, pronotum, prothorax, scutellum, ventral side of mesothorax and metathorax, abdomen, and legs orange; clypeus and mouthparts black; antennae black except 1^st^ segment paler; tibiae slightly dark orange, tarsi black; basal area of elytra orange, middle to apical area black with an oval white spot.

Vertex slightly convex, with transverse wrinkles, punctures obvious, space between punctures almost equal to diameter of punctures, each puncture with a seta; frontal tubercle obvious, not deeply divided by ecdysial suture, triangular, glabrous and with several large punctures near frontal area; antennae longer than half of body, 1^st^ segment arc-shaped, length ratio of 2^nd^ and 3^rd^ segment 19: 18; length ratio of 4^th^ and the combination of 2^nd^ and 3^rd^ 2: 1.

Pronotum transverse, pronotum around 1.6 times as broad as long; disc slightly convex, glabrous, shallowly depressed on each side, surface with irregular strong and fine punctures, each puncture with short seta.

Scutellum triangular, smooth and impunctate.

Elytra about 1.5 times as long as broad, basal part wider than pronotum; humeral angle obvious; two types of punctures in elytra: space between large punctures about 3 times as wide as diameter of puncture, small punctures irregularly distributed; epipleuron strongly narrowed after basal 1/3 and disappearing at the beginning of apex.

Ventral surface of mesothorax and metathorax covered with long setae. 1^st^ segment of hind tarsi 1.9 times as long as remaining segments combined. Anterior coxal cavities open.

**Male.** Last ventrite of male with trilobite concavities. The median apical lobe of the last sternite around twice as broad as long (Fig. 5). Aedeagus very slender, almost parallel-sided from base to middle, suddenly narrowed before 1/2 part, rounded at apex, slightly curved towards ventral side (Fig. 9). Tectum extends almost to apex of aedeagus (Fig. 8).

**Female.** Last ventrite of female with very slight concavities. Spermathecal cornu slender, curved almost vertical, middle part short, curved, nodulus middle narrow. Ventral part of bursa sclerites slender, slightly undulate at outer side, dorsal pair slender, pointed at apex.

##### Etymology.

The specific epithet *albipunctatus*, -*a*, -*um* (meaning ‘white-spotted’) is a New Latin adjective formed from the Latin adjective *albus*, -*a*, -*um* (‘white’) and the New Latin adjective *punctatus*, -*a*, -*um* (‘punctate’, ‘marked by spots or punctures’); it refers to the large white spots on the elytra of this species.

##### Distribution.

China: Guangxi.

##### Diagnosis.

This species is similar to *M.postfasciata* Gressitt & Kimoto, 1963, but the latter has a smaller body with an obvious T-shaped black spot on each elytron, whereas *M.albipunctata* sp. nov. has a larger body with two separate large, white, round spots on each elytron.

**Figures 1–10.**
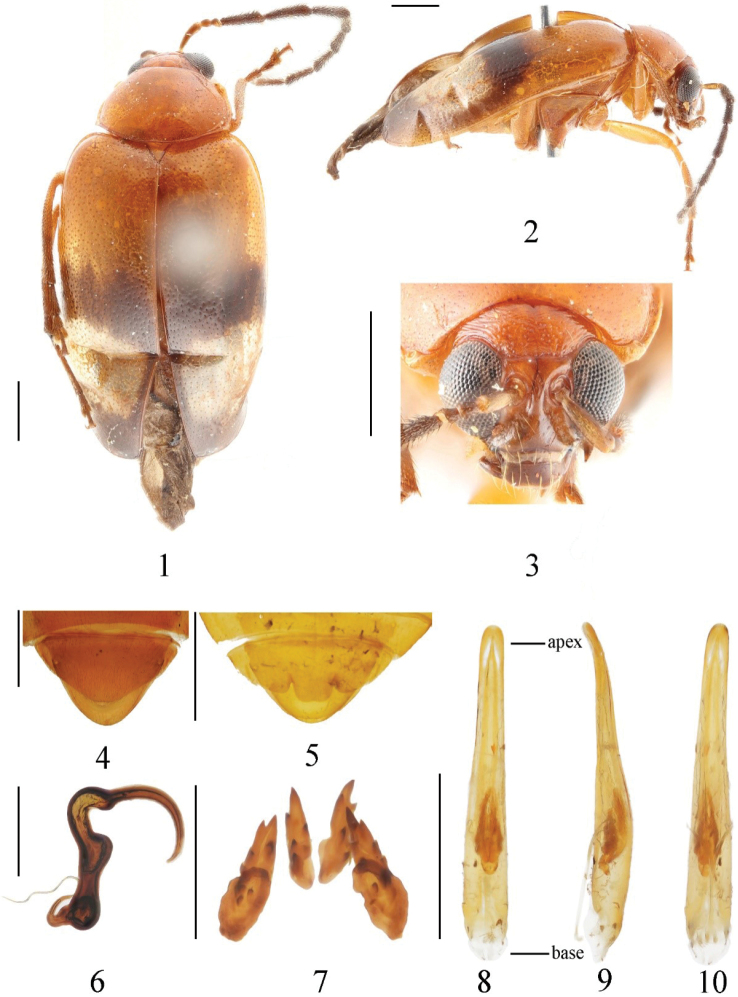
*Monoleptaalbipunctata* sp. nov. (holotype) **1** dorsal view **2** lateral view **3** frontal view **4** ventral view of 5^th^ ventrite, female **5** ditto, male **6** spermatheca **7** bursa sclerites **8** aedeagus, dorsal view **9** ditto, lateral view **10** ditto, ventral view. Scale bars: 1 mm (**1–5, 8–10**); 0.5 mm (**6, 7**).

#### Monolepta alticola sp. nov.

Taxon classificationAnimaliaColeopteraChrysomelidae

AA07D5B4-C012-5AD8-8A7F-FF97EC4B03A3

http://zoobank.org/FE2246D1-7C30-42C5-9D58-2137498E3545

Figs 11–20

##### Type material.

***Holotype***: China • ♂; Yunnan, Zhongdian, Gezan; 3000 m; 3-VIII-2003 (IZAS). ***Paratypes***: China • 1♂; Yunnan, Zhongdian, Gezan; 3000 m; 3-VIII-2003 • 2♀♀; Yunnan, Lunan, Shilin; 1700 m; 9-VII-1956; Kryzhanovskiy leg. (IZAS).

##### Description.

Length: 2.5–3.5 mm, width: 1.5–2.0 mm. Holotype: length 3.5 mm, width 2.0 mm.

Vertex orange, frons yellow, mouthparts dark brown; antennae dark brown except segments 1–3 brown; dorsal and ventral side of prothorax, coxae of front legs, femora yellow; scutellum, elytra, ventral side of mesothorax, metathorax, middle and hind legs dark brown; tibiae and tarsi of front legs pale brown, apex of middle and hind legs pale yellow.

Vertex convex, punctures sparsely and irregularly distributed; frontal tubercle developed; antennae longer than half of body, 1^st^ segment arc-shaped, length ratio of 2^nd^ and 3^rd^ segment 16: 15, length ratio of 4^th^ segment and the combination of 2^nd^ and 3^rd^ 45: 31.

Pronotum transverse, around 1.6 times as broad as long; disc slightly convex, shallowly depressed on each side, punctures unapparent, sparsely distributed; space between punctures wider than diameter of punctures.

Scutellum triangular, smooth and impunctate. The elytron about 1.6 times as long as broad; basal part wider than pronotum, humeral angle obvious; punctures on elytra irregularly distributed, space between punctures about 3 times as diameter of punctures. Epipleuron strongly narrowed after basal 1/3 and disappearing at beginning of apex.

Ventral surface of mesothorax, metathorax, and abdomen covered with long hairs.

Width and length ratio of median apical lobe 1.1 (apex part width to length), 2.2 (basal part width to length) (Fig. 15). 1^st^ segment of hind tarsi about 1.7 times as long as remaining segments combined.

**Male.** Aedeagus slender, ratio of length and width around 5; greatest width in basal 1/3, and suddenly narrowed from basal 1/2 and parallel sided; apex slightly cuspidate. Tectum extends almost to the apex of aedeagus, cuspidate apically (Fig. 18).

**Female.** Last ventrite of female normal, male with trilobite concavities. Spermathecal cornu slender, apex slightly pointed, middle part short, curved, nodulus nearly spherical, large.

##### Etymology.

The specific epithet *alticola*, *altus* (meaning ‘living in high altitude’) is a Latin adjective and the Latin *col*, (‘lives’); it refers to the high-altitude habitat of this species.

##### Distribution.

China: Yunnan.

##### Diagnosis.

This species is similar to *M.schereri* Gressitt & Kimoto, 1963 and *M.epistomalis* Laboissière, 1935. The main differences are the following: the ventral side of the meso- and meta-thorax and the abdomen of *M.schereri* are yellowish brown, whereas these are dark brown in *M.alticola* sp. nov. *M.epistomalis* has a dark-brown head and a yellowish-brown abdomen, whereas *M.alticola* sp. nov. has a yellow head and a black abdomen, and with the aedeagus tapering towards its apex.

**Figures 11–20.**
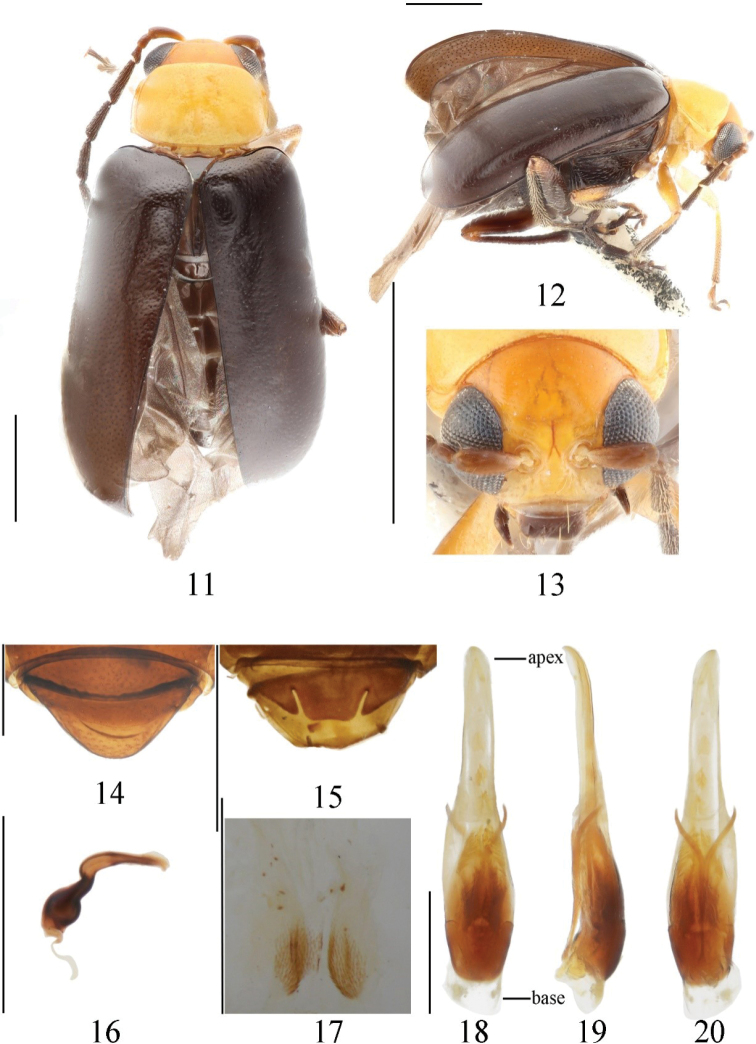
*Monoleptaalticola* sp. nov. (holotype) **11** dorsal view **12** lateral view **13** frontal view **14** ventral view of 5^th^ ventrite, female **15** ditto, male **16** spermatheca **17** bursa sclerites **18** aedeagus, dorsal view **19** ditto, lateral view **20** ditto, ventral view. Scale bars: 1 mm (**11–15, 18–20**); 0.5 mm (**16, 17**).

#### Monolepta bivittata sp. nov.

Taxon classificationAnimaliaColeopteraChrysomelidae

84AC06FF-ECE4-5647-B131-73CD105E6016

http://zoobank.org/B9A59187-352B-4171-8B08-B49BB8A1061D

Figs 21–27

##### Type material.

***Holotype***: China • ♂; Zhejiang, Taishun, Wuyanling Nature Reserve station by light; 800 m; 1-VIII-2005; Liu Ye leg. (IZAS). ***Paratypes***: China • 3♂♂; Zhejiang, Taishun, Wuyanling Nature Reserve station, at light; 800 m; 1-VIII-2005; Liu Ye leg. (IZAS).

##### Description.

Length: 3.0–3.6 mm, width: 1.5–1.7 mm. ***Holotype***: length 3.6 mm, width 1.7 mm.

Head, dorsal and ventral side of prothorax, and legs yellowish brown; mouthparts, scutellum, ventral side of mesothorax and metathorax black; antennae black, except segments 1–3 yellowish brown; elytra and abdomen pale yellow, basal and postmedian area of elytra with transverse black stripe.

Vertex convex, with sparsely distributed punctures; frontal tubercle developed, trapezoid, glabrous and without punctures; antennae reach half of body, 1^st^ segment arc-shaped, length ratio of segment 2^nd^ and 3^rd^ 15: 16, length ratio of 4^th^and combination of 2^nd^ and 3^rd^ 34: 31.

Pronotum about 1.5 times as broad as long; disc slightly convex, shallowly depressed on each side, punctures unapparent and sparsely distributed.

Scutellum triangular, smooth and impunctate. Elytron about 1.5 times as long as broad; basal part wider than pronotum, humeral angle obvious; punctures evenly distributed, space between punctures is about 2 times as diameter of puncture, each puncture with seta; epipleuron strongly narrowed after basal 1/3, disappearring at beginning of apex. Ventral side of mesothorax, metathorax and abdomen covered with long hairs.

**Male.** Last ventrite of male with trilobite concavities. Width and length ratio of median apical lobe 1.4 (apex width to length), 1.5 (basal width to length) (Fig. 24). Aedeagus: ratio of length to width around 4:3; gradually and slightly tapering from base to near apex then abruptly constricted in distal 1/5 in lateral view; apex rounded and slightly pointed (Figs 25, 27). Tectum broad, long, reaching to apex of aedeagus (Fig. 25).

##### Etymology.

The specific epithet *bivittatus*, -*a*, -*um* (meaning ‘bivittate’, ‘having two bands or stripes or vittae’) is a New Latin adjective formed from the Latin prefix *bi*- (a shortened form of *bis*, ‘twice’) and the Latin adjective *vittatus*, -*a*, -*um* (‘banded’); it refers to the two transverse black stripes on the elytra of this species, a character which distinguishes this species from all other species in the genus.

##### Distribution.

China: Zhejiang.

##### Diagnosis.

This species is similar to *M.leechi* Jacoby, 1890, *M.maana* Gressitt & Kimoto, 1963, and *M.liui* Gressitt & Kimoto, 1963. The main differences are the following: the abdomen of *M.leechi* is black and the apex of the aedeagus is sharp, whereas the abdomen of *M.bivittata* sp. nov. is pale yellow and the apex of the aedeagus is blunt. The space between the punctures on the elytra of *M.maana* is equal to the diameter of the punctures, whereas in *M.bivittata* sp. nov., it is about twice the diameter of the punctures. The mid- and hind-legs of *M.liui* are dark brown, whereas the legs of *M.bivittata* sp. nov. are yellowish brown.

**Figures 21–27.**
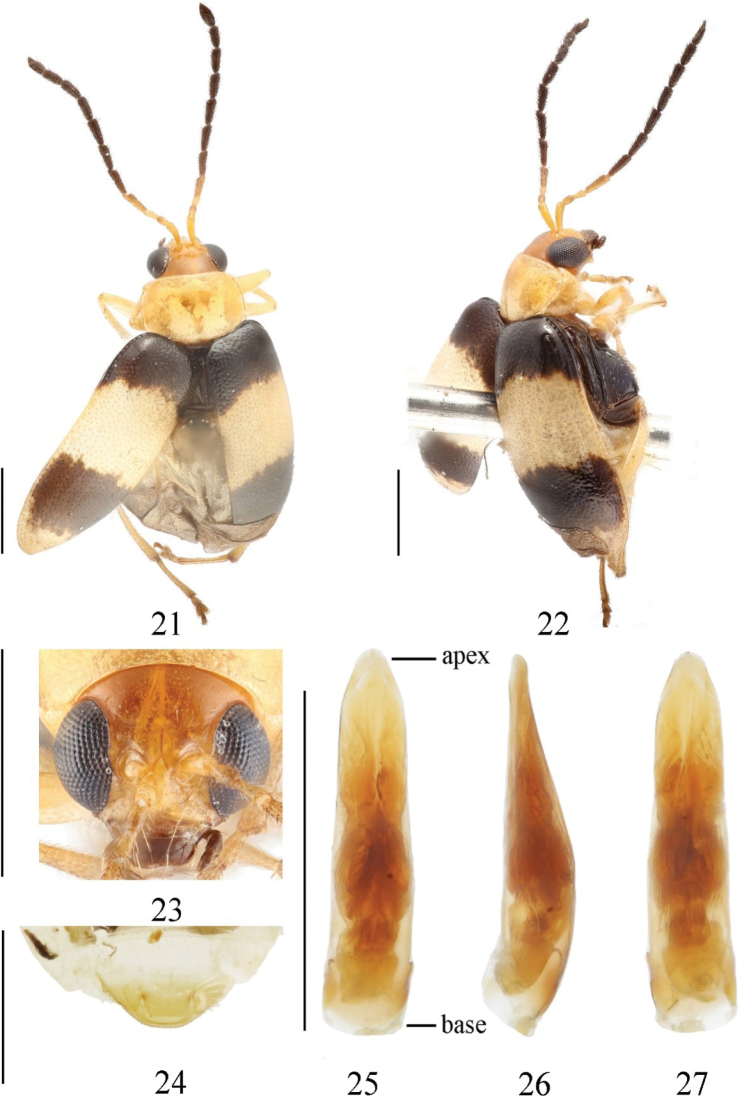
*Monoleptabivittata* sp. nov. (holotype) **21** dorsal view **22** lateral view **23** frontal view **24** ventral view of 5^th^ ventrite, male **25** aedeagus, dorsal view **26** ditto, lateral view **27** ditto, ventral view. Scale bars: 1 mm.

#### Monolepta mengsongensis sp. nov.

Taxon classificationAnimaliaColeopteraChrysomelidae

020F9BA2-730C-5CAF-BA94-CC408385DE09

http://zoobank.org/BCF69DF0-2A5A-49AC-BB27-36D0D561EC9D

Figs 28–34

##### Type material.

***Holotype***: China • ♂; Yunnan, Menglong, Banna, Mengsong; 1600 m; 27-IV-1958; Shu-yong Wang leg. (IZAS). ***Paratype***: China • 1♂; Yunnan, Menglong, Banna, Mengsong; 1600 m; 27-IV-1958; Shu-yong Wang leg. (IZAS).

**Description.** Length: 5.5–6.5 mm, width 3–3.5 mm. ***Holotype***: length 6.5 mm, width 3.5 mm.

Head, dorsal and ventral side of prothorax, mesothorax, metathorax, and femora orange; mouthparts darker; antennae dark brown, 1^st^ segment pale; scutellum, tibiae, and tarsi black; a wide, transverse, pale, yellowish-brown stripe after middle part of elytra, which reaches middle sutures but not to lateral margins.

Vertex convex, with transverse wrinkle, punctures obvious and evenly distributed, space between punctures is about twice as diameter of punctures; frontal tubercle developed, deeply divided by ecdysial suture, not reaching compound eye, triangular, glabrous and with a few punctures; antennae reach apex of elytra, 1^st^ segment arc-shaped, 2^nd^ antennomere equal to 3^rd^, 4^th^ segment longer than sum of 2^nd^ and 3^rd^.

Pronotum about 1.5 times as broad as long; disc slightly convex, shallowly depressed on each side; punctures obvious, densely and irregularly distributed, space between punctures wider than diameter of punctures.

Scutellum triangular, smooth and impunctate. Elytron nearly 1.6 times as long as broad; basal part broader than pronotum, humeral angle obvious; punctures evenly distributed, space between punctures about 2–3 times diameter of punctures. Epipleura strongly narrowed after basal 1/3 and disappearing at beginning of apex. Ventral side of mesothorax, metathorax and abdomen covered with long hairs.

**Male.** Last ventrite of male with trilobite concavities. Width and length ratio of median apical lobe 0.76 (apex width to length), 1.3 (basal width to length) (Fig. 31). Aedeagus almost straight, parallel sided in apical 1/5, slightly widened in the middle part, narrowed after 1/3 of apex, rounded at apex, slightly curved towards ventral side (Fig. 33). Tectum not reaching apex of aedeagus, apex rounded (Fig. 32). 1^st^ segment of hind tarsi about 1.5 times as long as remaining segments combined.

##### Etymology.

This species is named after its type locality at Mengsong.

##### Distribution.

China: Yunnan.

##### Diagnosis.

This species resembles *M.leechi*, *M.liui*, and *M.lunata*, but the length of the antennae reaches half of the body in *M.leechi* and *M.liui*, whereas in this species the length of the antennae reaches the apex of elytra. *M.lunata* has a rounded spot on the elytra, but in the new species there is a transverse band. This species has a transverse stripe on each elytron and its antennae reach the apex of the elytra, which can easily distinguish it from other species of *Monolepta* with transverse stripes.

**Figures 28–34.**
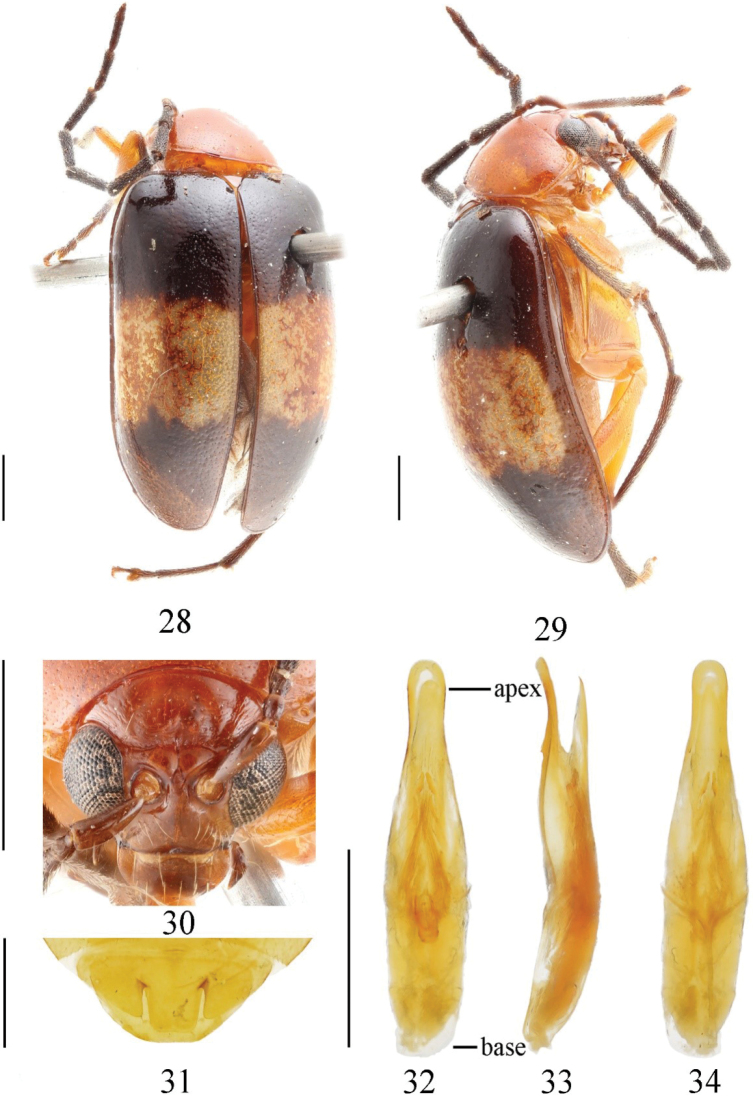
*Monoleptamengsongensis* sp. nov. (holotype) **28** dorsal view **29** lateral view **30** frontal view **31** ventral surface of 5^th^ ventrite, male **32** aedeagus, dorsal view **33** ditto, lateral view **34** ditto, ventral view. Scale bars: 1 mm.

#### Monolepta rubripennis sp. nov.

Taxon classificationAnimaliaColeopteraChrysomelidae

5D1CAEB1-ECD1-5592-A225-71553711CA29

http://zoobank.org/9B3D7F14-3838-450E-959E-1274765678C5

Figs 35–44

##### Type material.

***Holotype***: China • ♂; Sichuan, Mount Emei, Baoguo temple; 550–750 m; 2-VI-1957; Ke-ren Huang leg. (IZAS). ***Paratypes***: China • 2♀♀; Hunan, Guiding, Sidu, Xinlong village; 12-VII-2008; Hong-bin Liang leg. • 1♂; Fujian, Chongan, Xing village, Sangang; 740 m; 4-VI-1960; Yong Zuo leg. • 1♀; Sichuan, Mount Emei, Baoguo temple; 550–750 m; Ke-ren Huang leg.; 2-VI-1957 • 1♀; Mount Emei; 28-II-1955; Ke-ren Huang leg. • 1♀; Sichuan, Mount Emei, Baoguo temple; 550–750 m; 29-V-1957; Zong-yuan Wang leg. (all IZAS).

##### Description.

Length: 4.5–5.5 mm, width 2.2–3.0 mm. ***Holotype***: length 5.5 mm, width 2.8 mm.

Head, pronotum, prothorax, and legs black; scutellum, elytra, mesothorax, metathorax, and abdomen orange to reddish brown. Basal 1/2 of hind femur orange.

Vertex slightly convex with transverse wrinkle visible only laterally, punctures sparsely and irregularly distributed; frontal tubercle developed, deeply divided by ecdydial suture, triangular, not very glabrous and with many wrinkles on; antennae reach half of the body, 1^st^ segment arc-shaped, length ratio of segment 2^nd^ and 3^rd^ 19:21, length ratio of 4^th^ and the combination of 2^nd^ and 3^rd^ 23:18.

The pronotum is about 1.7 times as broad as long; disc slightly convex, shallowly depressed on each side; surface with irregular strong punctures, densely distributed near anterior margin, sparsely near basal margin. Anterior coxal cavities open.

Scutellum triangular, smooth and impunctate. Elytra is about 1.4 times as long as broad; basal part wider than pronotum, humeral angle obvious; punctures on elytra evenly distributed, with very short seta, space between punctures about 2–4 times as diameter of punctures; epipleuron strongly narrowed after basal 1/3 and disappearing at the beginning of apex. Ventral side of mesothorax, metathorax and abdomen glabrous, covered with longhairs.

The width and length ratio of median apical lobe is 1.2 (apex width to length), 2.3 (basal width to length) (Fig. 39). The 1^st^ segment of hind tarsi is about 1.5 times as long as remainder combined.

**Male.** Last ventrite of male with trilobite concavities. Aedeagus very slender and evenly narrowing from base to apex, apex rounded with a small cuspidate process. Tectum not reaching the apex of aedeagus, acute angle apex and curved towards ventral side (Fig. 43).

**Female.** Last ventrite of female normal. Spermathecal cornu curved strongly, middle part short, curved, very slender, nodulus small, nearly spherical. Ventral part of bursa sclerites fusiform, dorsal pair triangular, pointed at apex.

##### Etymology.

The specific epithet *rubripennis*, *rubripenne* (meaning ‘having red feathers or wings’) is a New Latin adjective formed from the Latin adjective *ruber*, *rubra*, -*um* (‘red’) and the Latin noun *penna*, -*ae* (‘feather’, ‘wing’); it refers to the red elytra of this species.

##### Distribution.

China: Hunan, Fujian, Sichuan.

##### Diagnosis.

This species is similar to *M.rufipennis* Jacoby, 1899 and *M.langbianica* Kimoto, 1989. The main differences are the following: *M.rubripennis* sp. nov. has. an orange abdomen and black antennae, whereas *M.rufipennis* has a black abdomen and yellow antennae, and *M.langbianica* has yellowish-brown antennae and a yellowish-brown abdomen.

**Figures 35–44.**
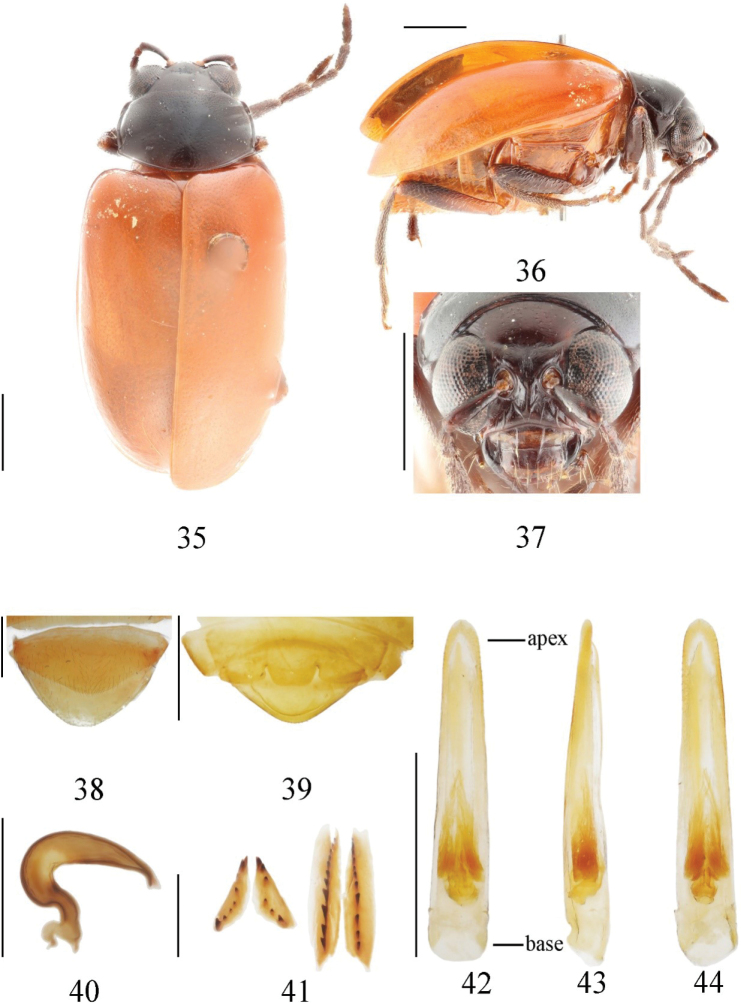
*Monoleptarubripennis* sp. nov. (holotype) **35** dorsal view **36** lateral view **37** frontal view **38** ventral view of 5^th^ ventrite, female **39** ditto, male **40** spermatheca **41** bursa sclerites **42** aedeagus, dorsal view **43** ditto, lateral view **44** ditto, ventral view. Scale bars: 1 mm (**35–39, 42–44**); 0.5 mm (**40, 41**).

## Supplementary Material

XML Treatment for Monolepta

XML Treatment for Monolepta albipunctata

XML Treatment for Monolepta alticola

XML Treatment for Monolepta bivittata

XML Treatment for Monolepta mengsongensis

XML Treatment for Monolepta rubripennis

## Supplementary materials

Supplementary material 1 Appendix S1. *Monolepta* checklist Data type catalogue and habitus Explanation note The file contains the catalogue of Chinese *Monolepta* with third antennal segment longer than second are denoted by Wagner. Also provide the habitus of most of these species. https://binary.pensoft.net/file/578893This dataset is made available under the Open Database License (http://opendatacommons.org/licenses/odbl/1.0/). The Open Database License (ODbL) is a license agreement intended to allow users to freely share, modify, and use this Dataset while maintaining this same freedom for others, provided that the original source and author(s) are credited.Qi-long Lei, Rui-e Nie, Xing-ke Yang
