# Supplementary material for: Five new species of the leaf-beetle genus Monolepta Chevrolat (Coleoptera, Chrysomelidae, Galerucinae) from China
Source: Zookeys. 2021 Aug 18;1056:35–57. doi: 10.3897/zookeys.1056.65335 (PMC8387305; doi:10.3897/zookeys.1056.65335)
Supplement: Supplementary material 1 — Appendix S1. Monolepta checklist [file zookeys-1056-035-s001.docx]

**Appendix S1. Catalogue of Chinese *Monolepta***

**Note. Species with third antennal segment longer than second are denoted by Wagner**

*Abbreviation of words in this study:*

Type locality: TL.

Type deposition: TD.

*For this catalogue, the type species were deposited in the following museums.*

**BMNH** The Natural History Museum, London, UK

**BPBM** Bishop Museum Honolulu, Hawaii, USA

**BRH** Biohistory Research Hall, Takatsuki, Osaka, Japan

**CAS** California Academy of Sciences, San Francisco, California, USA

**EIHU** Institute of Systematic Entomology, Hokkaido University, Japan

**IZAS** Institute of Zoology, Chinese Academy of Sciences, Beijing, China

**KUEC** Kyushu University, Fukuoka, Japan

**KUTJ** Kagawa University, Entomology laboratory, Takamatsu-shi, Japan

**LM** Lev Medvedev’s collection

**MCZC** Museum of Comparative Zoology, Cambridge, Harvard University, Massachusetts, USA

**MNHN** Muséum National d'Histoire Naturelle, Paris, France

**NHRS** Naturhistoriska riksmuseet, Stockholm, Sweden

**NMEG** Naturkundemuseum, Erfurt, Germany

**SDEI** Senckenberg Deutsches Entomologisches Institut, Müncheberg, Germany

**SMNS** Staatliches Museum fww.naturkunde, Stuttgart, Germany

**USNM** National Museum of Natural History, [formerly, United States National Museum], Washington D.C., USA

**ZMHB** Museum für Naturkunde der Humboldt-Universität, Berlin, Germany

**ZMUM** Moscow State University, Moscow, Russia

***Monolepta aglaonemae* Gressitt & Kimoto, 1963 (Fig. A1)**

*Monolepta aglaonemae* Gressitt & Kimoto, 1963: 607. **TL** China: Hainan. **TD** CAS.

**Distribution** China: Fujian, Guangdong, Hainan.

**Host plant** *Aglaonema* sp.

***Monolepta alnivora* Chen, 1976 (Fig. A2)**

*Monolepta alnivora* Chen, 1976: 205. **TL** China: Xizang. **TD** IZAS.

**Distribution** China: Xizang.

**Host plant** *Alnus cremastogyne*.

***Monolepta annamita* (Laboissière, 1935)**

*Monolepta annamita* Laboissière, 1935: 143 (new name for *Luperodes ferruginea* Allard, 1889). **TL** Vietnam. **TD** MNHN.

*Luperodes ferruginea* Allard, 1889: 311 (nec Karsch, 1882).

**Distribution** China: Taiwan. Vietnam, Laos, Thailand.

***Monolepta arundinariae* Gressitt & Kimoto, 1963 (Fig. A3)**

*Monolepta arundinariae* Gressitt & Kimoto, 1963: 609. **TL** China: Jiangxi. **TD** CAS.

**Distribution** China: Henan, Jiangsu, Hubei, Jiangxi, Hunan, Fujian, Guangxi, Sichuan.

**Host plants** Gramineae, *Arundinaria* sp.

***Monolepta asahinai* Chûjô, 1962**

*Monolepta asahinai* Chûjô, 1962: 116. **TL** China: Taiwan. **TD** KUTJ.

**Distribution** China: Taiwan.

***Monolepta bacboensis* Medvedev, 2012**

*Monolepta bacboensis* Medvedev, 2012: 494. **TL** China: Yunnan. **TD** LM.

Distribution China: Yunnan; Vietnam.

***Monolepta brittoni* Gressitt & Kimoto, 1963 (Fig. A4)**

*Monolepta brittoni* Gressitt & Kimoto, 1963: 604, 611. **TL** China: Hainan. **TD** CAS.

**Distribution** China: Hainan.

***Monolepta capitata* Chen, 1942 (Fig. A5)**

*Monolepta capitata* Chen, 1942: 58. **TL** China: Sichuan. **TD** IZAS.

**Distribution** China: Hubei, Hunan, Fujian, Sichuan.

**Host plant** *Rubus* sp.

***Monolepta cavipennis* Baly, 1878 (Fig. A6)**

*Monolepta cavipennis* Baly, 1878: 459. **TL** India. **TD** BMNH.

*Luperodes albofasciata* Allard, 1889: 310. Synonymized by Kimoto (1989: 127).

*Diacantha fasciata* Allard, 1889: 318, 323. Synonymized by Kimoto (1989: 127).

*Candezea trifasciata* Jacoby, 1900: 139 (nec Jacoby, 1896). Synonymized by Bryant (1923: 146).

*Monolepta siamensis* Weise, 1915: 177 (new name for *Candezea* *trifasciata* Jacoby, 1900).

**Distribution** China: Guangdong, Hainan, Hong Kong, Guangxi, Yunnan. Vietnam, Laos, Cambodia, Thailand, India.

***Monolepta cheni* Beenen, 2008 (Fig. A7)**

*Monolepta cheni* Beenen, 2008: 65 (new name for *Monolepta straminea* Chen, 1976). **TL** China: Xizang. **TD** IZAS.

*Monolepta straminea* Chen, 1976: 206 (nec Harold, 1880).

**Distribution** China: Xizang.

***Monolepta chinkinyui* Kimoto, 1996**

*Monolepta chinkinyui* Kimoto, 1996: 38. **TL** China: Taiwan. **TD** BRH.

**Distribution** China: Taiwan.

***Monolepta discalis* Gressitt & Kimoto, 1963 (Fig. A8)**

*Monolepta discalis* Gressitt & Kimoto***,*** 1963: 612. **TL** China: Yunnan. **TD** BSBM.

**Distribution** China: Gansu, Zhejiang, Guizhou, Yunnan.

***Monolepta epistomalis* Laboissière, 1934 (Fig. A9)**

*Monolepta epistomalis* Laboissière, 1934: 9. **TL** China: Gansu. **TD** NHRS.

**Distribution** China: Gansu, Zhejiang, Hunan.

***Monolepta erythrocephala* (Baly, 1878) (Fig. A10)**

*Luperodes erythrocephala* Baly, 1878: 380. **TL** Pakistan. **TD** BMNH.

*Monolepta erythrocephala* Maulik, 1936: 430.

**Distribution** China: Sichuan. India, Nepal, Bhutan, Pakistan.

***Monolepta eunicia* Maulik, 1936 (Fig. A11)**

*Monolepta eunicia* Maulik, 1936: 395. **TL** India. **TD** BMNH.

**Distribution** China: Sichuan, Guizhou. Myanmar, India.

***Monolepta flavovittata* Chen, 1942 (Fig. A12)**

*Monolepta flavovittata* Chen, 1942: 61. **TL** China: Hebei. **TD** IZAS.

**Distribution** China: Gansu, Shaanxi, Hebei, Hubei, Fujian. Vietnam.

***Monolepta gracilipes* Chûjô, 1938 (Fig. A13)**

*Monolepta gracilipes* Chûjô, 1938: 145. **TL** China: Taiwan. **TD** TARI., SDEI. Synonymized with *Monolepta pallidula* (Baly, 1874) by Kimoto (1969: 47).

*Monolepta gracilipes* Lee, 2009: 16. Removed from synonymy *Monolepta pallidula* (Baly, 1874).

**Distribution** China: Taiwan.

***Monolepta hongkongense* Kimoto, 1967**

*Monolepta hongkongense* Kimoto, 1967: 61. **TL** China: Hong Kong. **TD** KUEC.

**Distribution** China: Hong Kong.

***Monolepta horni* Chûjô, 1938 (Fig. A14)**

*Monolepta horni* Chûjô, 1938: 146. **TL** China: Taiwan. **TD** SDEI, TARI.

**Distribution** China: Taiwan.

***Monolepta hupehensis* Gressitt & Kimoto, 1963 (Figs. A15, 16)**

*Monolepta hupehensis* Gressitt & Kimoto, 1963: 604, 615. **TL** China: Hubei. **TD** IZAS.

**Distribution** China: Hebei, Hubei.

**Monolepta indochinensis Medvedev, 1999**

Monolepta indochinensis Medvedev, 1999: 184**.** **TL** Vietnam. **TD** LM, NMEG, SMNS.

**Distribution** China: Yunnan. Vietnam, Thailand.

***Monolepta kuroheri* Kimoto, 1966 (Fig. A17)**

*Monolepta kuroheri* Kimoto, 1966: 31. **TL** China: Taiwan. **TD** KUEC.

**Distribution** China: Taiwan.

***Monolepta lauta* Gressitt & Kimoto, 1963 (Fig. A18)**

*Monolepta lauta* Gressitt & Kimoto, 1963: 604, 617. **TL** China: Hainan. **TD** CAS.

**Distribution** China: Hainan, Guizhou. Vietnam, Laos.

***Monolepta leechi* Jacoby, 1890 (Figs. A19, 20)**

*Monolepta leechi* Jacoby, 1890: 216. **TL** China: Hubei. **TD** MCZC.

**Distribution** China: Hubei, Fujian, Taiwan, Guangdong, Guizhou, Yunnan. Vietnam,

Laos, India, Nepal.

***Monolepta liui* Gressitt & Kimoto, 1963 (Figs. A21, 22)**

*Monolepta liui* Gressitt & Kimoto, 1963: 618. **TL** China: Yunnan. **TD** USNM.

**Distribution** China: Guizhou, Yunnan.

***Monolepta longicornis* (Jacoby, 1890)**

*Taphinella longicornis* Jacoby, 1890: 194. **TL** China: Hubei. **TD** BMNH.

*Arthrotus longicornis* Gressitt & Kimoto, 1963: 698.

*Monolepta longicornis* Wilcox, 1973: 552.

**Distribution** China: Hubei.

***Monolepta longitarsoides* Chûjô, 1938 (Figs. A23, 24)**

*Monolepta longitarsoides* Chûjô, 1938: 147. **TL** China: Taiwan. **TD** TARI

**Distribution** China: Zhejiang, Hubei, Jiangxi, Hunan, Fujian, Taiwan, Guangdong,

Hainan, Guangxi, Sichuan, Guizhou.

***Monolepta lunata* Gressitt & Kimoto, 1963 (Fig. A25)**

*Monolepta lunata* Gressitt & Kimoto, 1963: 620. **TL** China: Hainan. **TD** CAS.

**Distribution** China: Hainan. India.

***Monolepta maana* Gressitt & Kimoto, 1963 (Fig. A26)**

*Monolepta maana* Gressitt & Kimoto, 1963: 621. **TL** China: Fujian. **TD** BSBM.

**Distribution** China: Fujian.

***Monolepta mandibularis* Chûjô, 1962**

*Monolepta mandibularis* Chûjô, 1962: 120. **TL** China: Taiwan. **TD** KUTJ.

**Distribution** China: Taiwan.

***Monolepta meihuai* Lee, Tian & Staines, 2010**

*Monolepta meihuai* Lee, Tian & Staines, 2010: 306. China: Taiwan. **TD** TARI.

**Distribution** China: Taiwan.

***Monolepta meridionalis* Gressitt & Kimoto, 1963 (Fig. A27)**

*Monolepta meridionalis* Gressitt & Kimoto, 1963: 622. **TL** China: Guangdong. **TD** CAS.

**Distribution** China: Guangdong, Hainan.

***Monolepta minor* Chûjô, 1938 (Fig. A28)**

*Monolepta minor* Chûjô, 1938: 149. **TL** China: Taiwan. **TD** TARI.

*Monolepta seminigra* Chûjô, 1962: 118. Synonymized by Kimoto (1969: 50).

**Distribution** China: Taiwan. Japan.

***Monolepta minutissima* Chen, 1942 (Fig. A29)**

*Monolepta minutissima* Chen, 1942: 60. **TL** China: Guangxi. **TD** IZAS.

**Distribution** China: Guangxi.

***Monolepta nakanei* Kimoto, 1969**

*Monolepta nakanei* Kimoto, 1969: 52. **TL** China: Taiwan. **TD** KUEC.

**Distribution** China: Taiwan.

***Monolepta occifluvis* Gressitt & Kimoto, 1963 (Fig. A30)**

*Monolepta occifluvis* Gressitt & Kimoto, 1963: 624. **TL** China: Guangdong. **TD** CAS.

**Distribution** China: Guangdong, Guangxi.

***Monolepta ongi* Lee & Staines, 2010 (Fig. A31)**

*Monolepta ongi* Lee & Staines, 2010: 538. **TL** China: Taiwan. **TD** TARI.

**Distribution** China: Taiwan.

***Monolepta ovatula* Chen, 1942 (Fig. A32)**

*Monolepta ovatula* Chen, 1942: 60. **TL** China: Guangxi. **TD** IZAS.

**Distribution** China: Hubei, Jiangxi, Fujian, Guangxi, Guizhou.

**Host plants** Bambusoideae.

***Monolepta pallidula* (Baly, 1874) (Fig. A33)**

*Luperodes pallidulus* Baly, 1874: 187. **TL** Japan. **TD** BMNH.

*Monolepta pallidula*: Chûjô, 1938: 144.

**Distribution** China: Gansu, Henan, Anhui, Zhejiang, Hubei, Jiangxi, Hunan, Fujian, Taiwan, Guangdong, Hainan, Guangxi, Sichuan, Guizhou, Yunnan, Xizang. Korea Peninsula, Japan, Vietnam, Laos, Thailand.

**Host plants** *Populus euphratica*, *Styrax japonica*, Styracaceae, Bambusoideae.

***Monolepta palliparva* Gressitt & Kimoto, 1963 (Fig. A34)**

*Monolepta palliparva* Gressitt & Kimoto, 1963: 626. **TL** China: Guizhou. **TD** BSBM.

**Distribution** China: Jiangxi, Hainan, Guizhou, Yunnan.

***Monolepta parenthetica* Gressitt & Kimoto, 1963 (Fig. A35)**

*Monolepta parenthetica* Gressitt & Kimoto, 1963: 627. **TL** China: Hubei. **TD** BSBM.

**Distribution** China: Hubei, Sichuan.

***Monolepta quadricavata* Chen, 1976 (Fig. A37)**

*Monolepta quadricavata* Chen, 1976: 205. **TL** China: Xizang. **TD** IZAS.

**Distribution** China: Xizang. Nepal.

***Monolepta quadriguttata* (Motschulsky, 1860)**

*Luperodes* *quadriguttata* Motschulsky, 1860: 233. **TL** Russia. **TD** ZMUM.

*Monolepta quadriguttata*: Ogloblin, 1936: 315, 320, 434.

**Distribution** China: Heilongjiang, Ningxia, Gansu, Guangxi, Yunnan. Russia, Korea Peninsula, Japan.

**Host plants** Leguminosae, Cruciferae, Cannabinaceae.

***Monolepta rufofulva* Chûjô, 1938 (Fig. A38)**

*Monolepta rufofulva* Chûjô, 1938: 149. **TL** China: Taiwan. **TD** TARI.

**Distribution** China: Taiwan, Sichuan, Yunnan.

***Monolepta sasajii* Kimoto, 1969 (Fig. A39)**

*Monolepta sasajii* Kimoto, 1969: 53. **TL** China: Taiwan. **TD** KUEC.

**Distribution** China: Taiwan.

***Monolepta sauteri* Chûjô, 1935 (Fig. A40)**

*Monolepta sauteri* Chûjô, 1935: 173. **TL** China: Taiwan. **TD** SDEI, TARI.

**Distribution** China: Fujian, Taiwan, Guangdong, Hainan, Guangxi, Guizhou, Yunnan.

***Monolepta schereri* Gressitt & Kimoto, 1963 (Fig. A41)**

*Monolepta schereri* Gressitt & Kimoto, 1963: 630. **TL** China: Hubei. **TD** CAS.

**Distribution** China: Hubei, Guizhou.

***Monolepta selmani* Gressitt & Kimoto, 1963 (Fig. A42)**

*Monolepta selmani* Gressitt & Kimoto, 1963: 631. **TL** China: Hubei. **TD** CAS.

**Distribution** China: Gansu, Zhejiang, Hubei, Hunan, Guizhou, Yunnan.

***Monolepta semenovi* Ogloblin, 1936 (Fig. A43)**

*Monolepta semenovi* Ogloblin, 1936: 318, 434. **TL** China: Sichuan. **TD** ZMUM.

**Distribution** China: Guizhou, Sichuan. Russia.

**Monolepta severini (Jacoby, 1896)**

Luperus severini Jacoby, 1896: 276. **TL** India.

Monolepta severini Kimoto, 2001: 42.

**Distribution** China: Yunnan. Nepal, India, Burma, Thailand, Laos, Vietnam.

***Monolepta sexlineata* Chûjô, 1938 (Fig. A44)**

*Monolepta sexlineata* Chûjô, 1938: 150. **TL** China: Taiwan. **TD** SDEI.

*Monolepta duvivieri* Jacoby, 1904: 404 (nec Jacoby, 1897).

*Monolepta lineata* Weise, 1915: 117 (nec Karsch, 1882).

*Monolepta madrasensis* Wilcox, 1973: 562. (new name for *Monolepta duvivieri* Jacoby, 1904 and for *Monolepta lineata* Weise, 1915). Synonymized by Kimoto (1989: 150).

**Distribution** China: Jilin, Hebei, Shanxi, Shaanxi, Gansu, Fujian, Taiwan, Guangdong, Hainan, Guangxi, Yunnan. Vietnam, Laos, Cambodia, Thailand, India, Nepal, Bhutan, Sri Lanka.

**Host plant** *Saccharum officinarum*.

***Monolepta shaowuensis* Gressitt & Kimoto, 1963 (Fig. A45)**

*Monolepta shaowuensis* Gressitt & Kimoto, 1963: 632. **TL** China: Fujian. **TD** BSBM.

**Distribution** China: Hubei, Jiangxi, Hunan, Fujian, Guangdong.

***Monolepta signata* (Oliver, 1808) (Fig. A46)**

*Galeruca signata* Olivier, 1808: 665. **TL** China: Hong Kong. **TD** MNHN.

*Crioceris neglecta* Sahlberg, 1829: 29. Synonymized by Maulik (1936: 410).

*Luperodes hieroglyphicus* Motschulsky, 1858: 104. Synonymized by Wagner (2012: 210).

*Luperodes quadripustulatus* Motschulsky, 1858: 105. Synonymized by Maulik (1936: 410).

*Monolepta elegantula* Boheman, 1859: 183. Synonymized by Weise (1913: 229).

*Luperodes dorsalis* Motschulsky, 1866: 415. Synonymized by Wagner (2012: 210).

*Luperodes quadriguttata* Fairmaire, 1887: 333. Synonymized by Weise (1913: 229).

*Monolepta signata* Jacoby, 1889: 229.

*Monolepta biarcuata* Weise, 1889: 632. Synonymized by Weise (1922: 104).

*Monolepta picturata* Jacoby, 1896: 292. Synonymized by Wagner (2012: 210).

*Monolepta simplex* Weise, 1913: 229. Synonymized by Weise (1924: 168).

*Monolepta signata* ab. *belmonti* Piton, 1943: 138. Synonymized by Wilcox (1973: 534).

*Monolepta signata* ab. *bipunctata* Piton, 1943: 138. Synonymized by Wilcox (1973: 533).

*Monolepta signata* ab. *coalita* Piton, 1943: 138. Synonymized by Wilcox (1973: 533).

*Monolepta signata* ab. *lutea* Piton, 1943: 138. Synonymized by Wilcox (1973: 533).

*Monolepta signata* ab. *pici* Piton, 1943: 138. Synonymized by Wilcox (1973: 533).

*Monolepta signata* ab. *sexmaculata* Piton, 1943: 138. Synonymized by Wilcox (1973: 534).

*Monolepta signata* ab. *yunnanensis* Piton, 1943: 138. Synonymized by Wilcox (1973: 534).

**Distribution** China: Heilongjiang, Jilin, Liaoning, Inner Mongolia, Gansu, Hebei, Shanxi, Shaanxi, Henan, Zhejiang, Hubei, Jiangxi, Hunan, Fujian, Taiwan, Guangdong, Hong Kong, Hainan, Guangxi, Sichuan, Guizhou, Yunnan, Xizang. Mongolia, Russia, Korea Peninsula, Japan, Vietnam, Laos, Thailand, Cambodia, India, Myanmar, Nepal, Bhutan, Sri Lanka, Philippines, Malaysia, Singapore, Indonesia, New Guinea, Australia.

**Host plants** Leguminosae, *Gossypium* sp., *Zea mays*, *Arachis hypogaea*, *Salix* sp., *Viburnum* sp., *Rubus sp*., *Pyracantha crenulata.*

***Monolepta subapicalis* Gressitt & Kimoto, 1963 (Fig. A47)**

*Monolepta subapicalis* Gressitt & Kimoto, 1963: 634. **TL** China: Sichuan. **TD** CAS.

**Distribution** China: Shaanxi, Gansu, Hubei, Hunan, Fujian, Guizhou. Vietnam, Bhutan.

***Monolepta subrubra* Chen, 1942 (Fig. A48)**

*Monolepta subrubra* Chen, 1942: 57. **TL** China: Shaanxi. **TD** IZAS.

**Distribution** China: Shaanxi, Fujian.

***Monolepta takizawai* Kimoto, 1996**

*Monolepta takizawai* Kimoto, 1996: 39. **TL** China: Taiwan. **TD** EIHU.

**Distribution** China: Taiwan.

**Monolepta weigeli Medvedev, 2012**

Monolepta weigeli Medvedev, 2012: 494. **TL** China: Yunnan. **TD** NMEG.

**Distribution** China: Yunnan.

***Monolepta wilcoxi* Gressitt & Kimoto, 1965 (Fig. A49)**

*Monolepta antennalis* Gressitt & Kimoto, 1963: 605 (nec Lea, 1923). **TL** China: Sichuan. **TD** BSBM.

*Monolepta wilcoxi* Gressitt & Kimoto, 1965: 801 (new name for *M. antennalis* Gressitt & Kimoto, 1963).

**Distribution** China: Sichuan, Guizhou.

***Monolepta xanthodera* Chen, 1942 (Fig. A50)**

*Monolepta xanthodera* Chen, 1942: 58. **TL** China: Shaanxi, Sichuan. **TD** IZAS.

**Distribution** China: Shaanxi, Gansu, Hubei, Hunan, Fujian, Taiwan, Sichuan, Guizhou, Yunnan, Xizang.

***Monolepta yama* Gressitt & Kimoto, 1965**

*Monolepta yama* Gressitt & Kimoto, 1965: 802 (new name for *M*. *monticola* Gressitt & Kimoto, 1963). **TL** China: Yunnan. **TD** BSBM.

*Monolepta monticola* Gressitt & Kimoto, 1963: 623 (nec Weise, 1915).

**Distribution** China: Henan, Shaanxi, Hubei, Gansu, Zhejiang, Hubei, Jiangxi, Hainan, Sichuan, Guizhou, Yunnan.

***Monolepta yunnanica* Gressitt & Kimoto, 1963 (Fig. A52)**

*Monolepta yunnanica* Gressitt & Kimoto, 1963: 637. **TL** China: Yunnan. **TD** ZMHB.

**Distribution** China: Hunan, Fujian, Sichuan, Yunnan.

***Monolepta zonalis* Gressitt & Kimoto, 1963 (Fig. A53)**

*Monolepta zonalis* Gressitt & Kimoto, 1963: 638. **TL** China: Yunnan. **TD** BSBM.

**Distribution** China: Yunnan. Vietnam, Laos.

**Antennae 3=2, but aedeagus different from the ‘true’ *Monolepta***

***Monolepta postfasciata* Gressitt & Kimoto, 1963 (Fig. A36)**

*Monolepta postfasciata* Gressitt & Kimoto, 1963: 628. **TL** China: Anhui. **TD** MCZC.

**Distribution** China: Gansu, Anhui, Hubei, Hunan, Fujian.

***Monolepta yaosanica* Chen, 1942 (Fig. A51)**

*Monolepta yaosanica* Chen, 1942: 59. **TL** China: Guangxi. **TD** IZAS.

**Distribution** China: Henan, Zhejiang, Hubei, Jiangxi, Fujian, Guangxi.

**
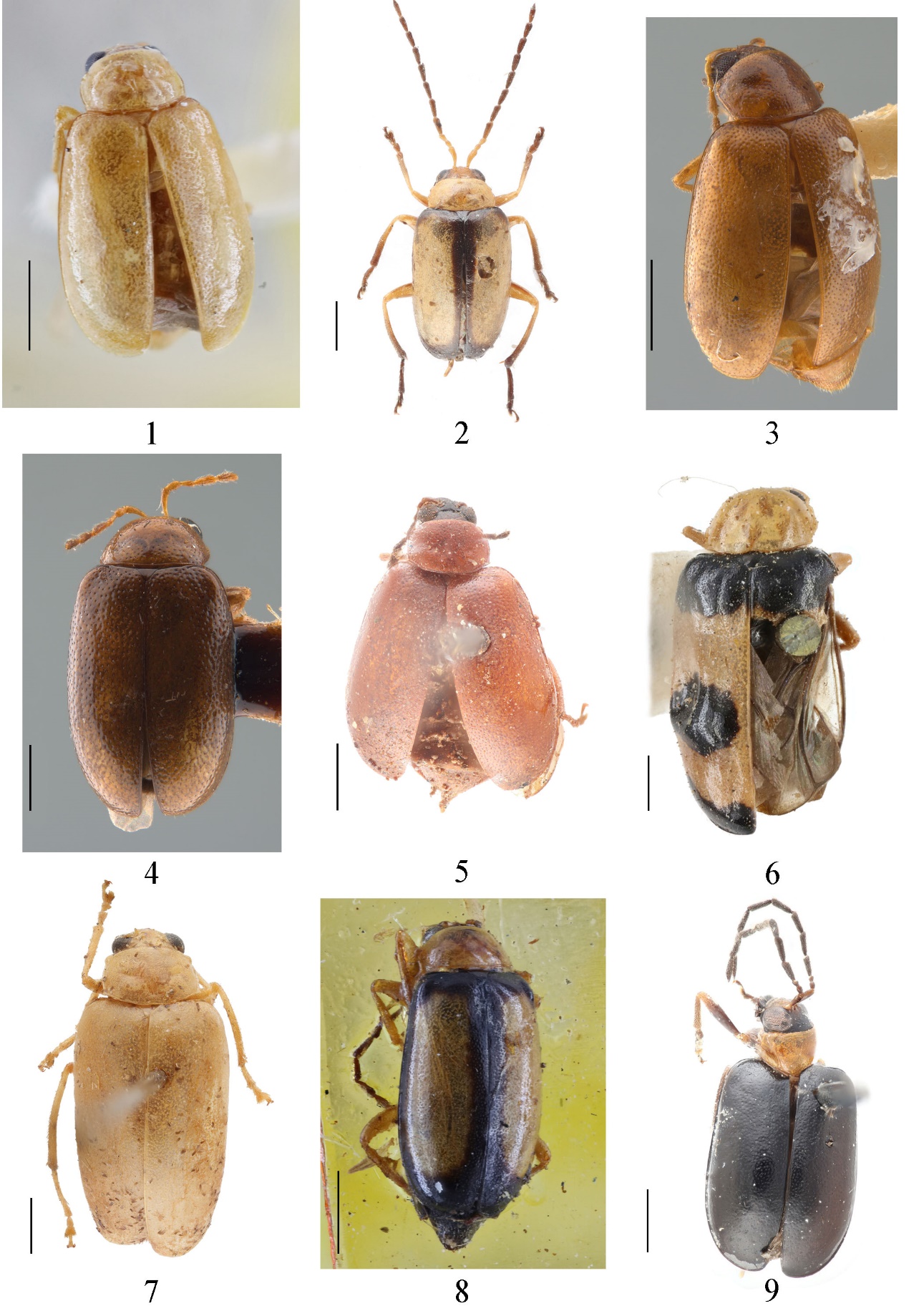
**

**Figures S1–9.** Habitus. **1** *M. aglaonemae* (Paratype, BMNH) **2** *M. alnivora* (identified species) **3** *M. arundinariae* (Holotype, CAS) **4** *M. brittoni* (Holotype, CAS) **5** *M. capitata* (Holotype, IZAS) **6** *M. cavipennis* (Paratype, BMNH) **7** *M. cheni* (Holotype, IZAS) **8** *M. discalis* (Paratype, BMNH) **9** *M. epistomalis* (identified species). Scale bar: 1 mm.

**
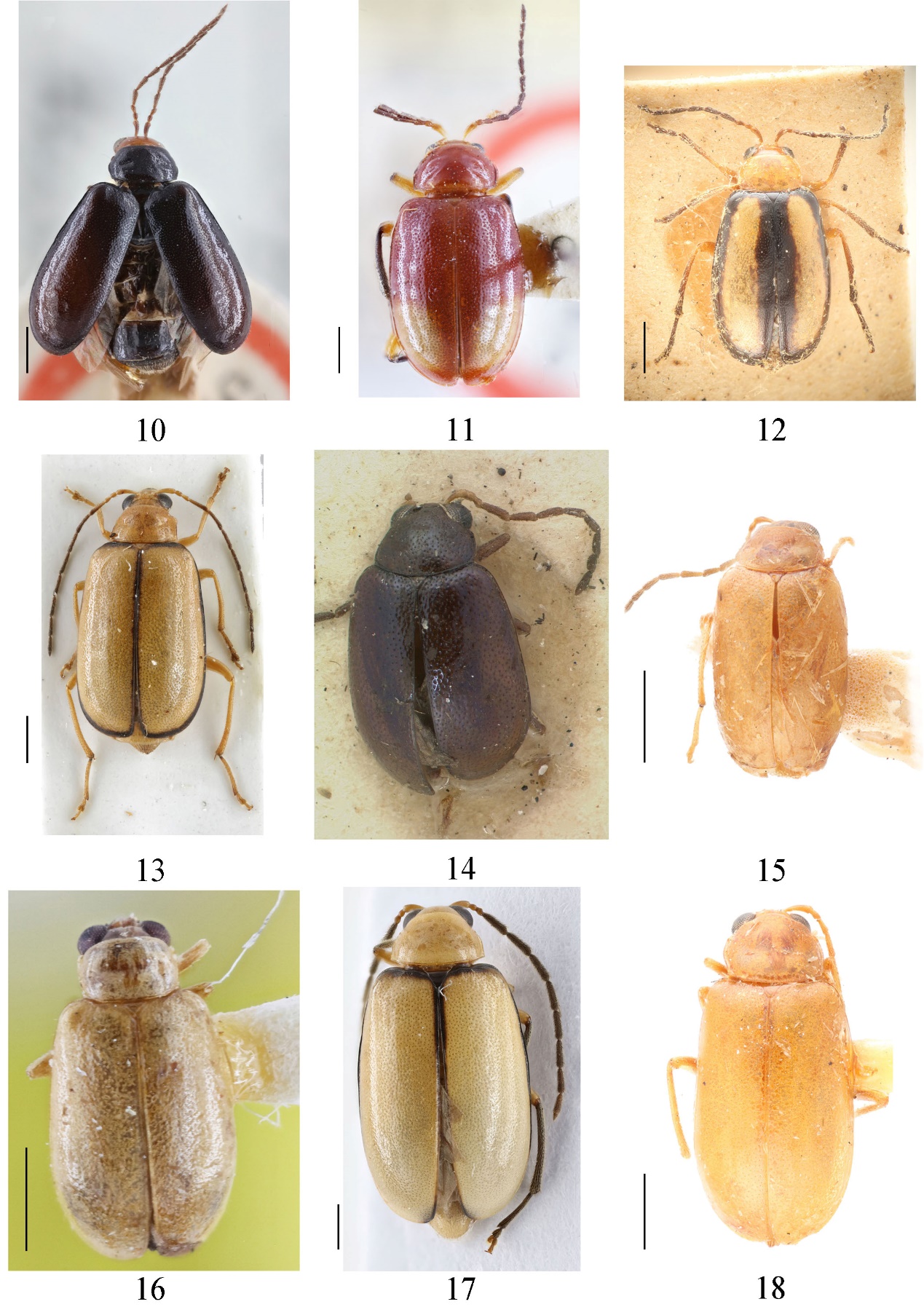
**

**Figures S10–18.** Habitus. **10** *M. erythrocephala* (Type, BMNH) **11** *M. eunicia* (Type, BMNH) **12** *M. flavovittata* (Holotype, IZAS) **13** *M. gracilipes* (identified species) **14** *M. horni* (Syntypus, NSNM) **15** *M. hupehensis* (Paratype, IZAS) **16** *M. hupehensis* (Paratype, BMNH) **17** *M. kuroheri* (identified species) **18** *M. lauta* (Paratype, IZAS). Scale bar: 1 mm.

**
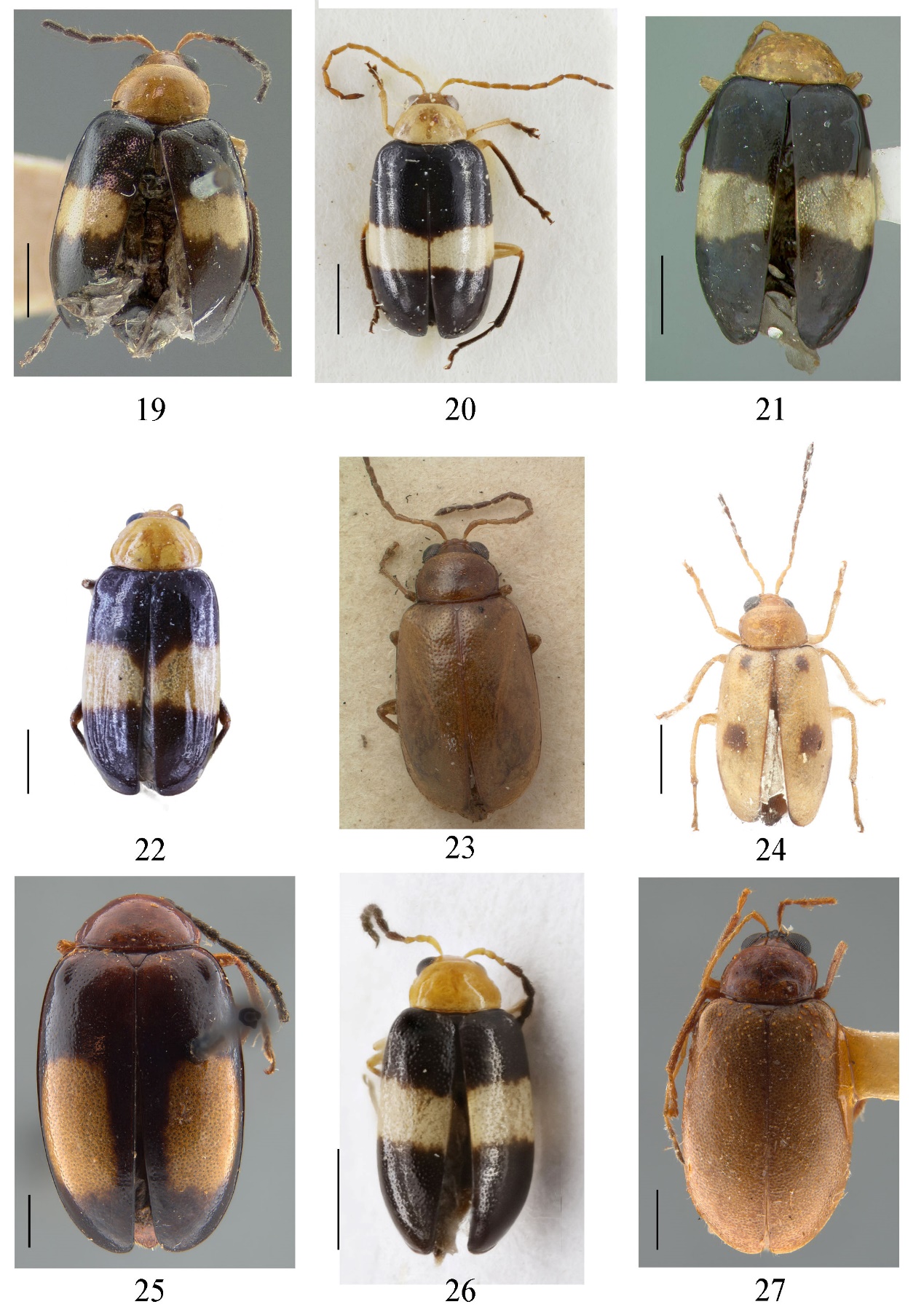
**

**Figures S19–27.** Habitus. **19** *M. leechi* (Holotype, MCZ) **20** *M. leechi* (identified species) **21** *M. liui* (Holotype, USNM) **22** *M. liui* (identified species) **23** *M. longitarsoides* (Syntypus, NSNM) **24** *M. longitarsoides* (identified species) **25** *M. lunata* (Holotype, CAS) **26** *M. maana* (identified species) **27** *M. meridionalis* (Holotype, CAS). Scale bar: 1 mm.

**
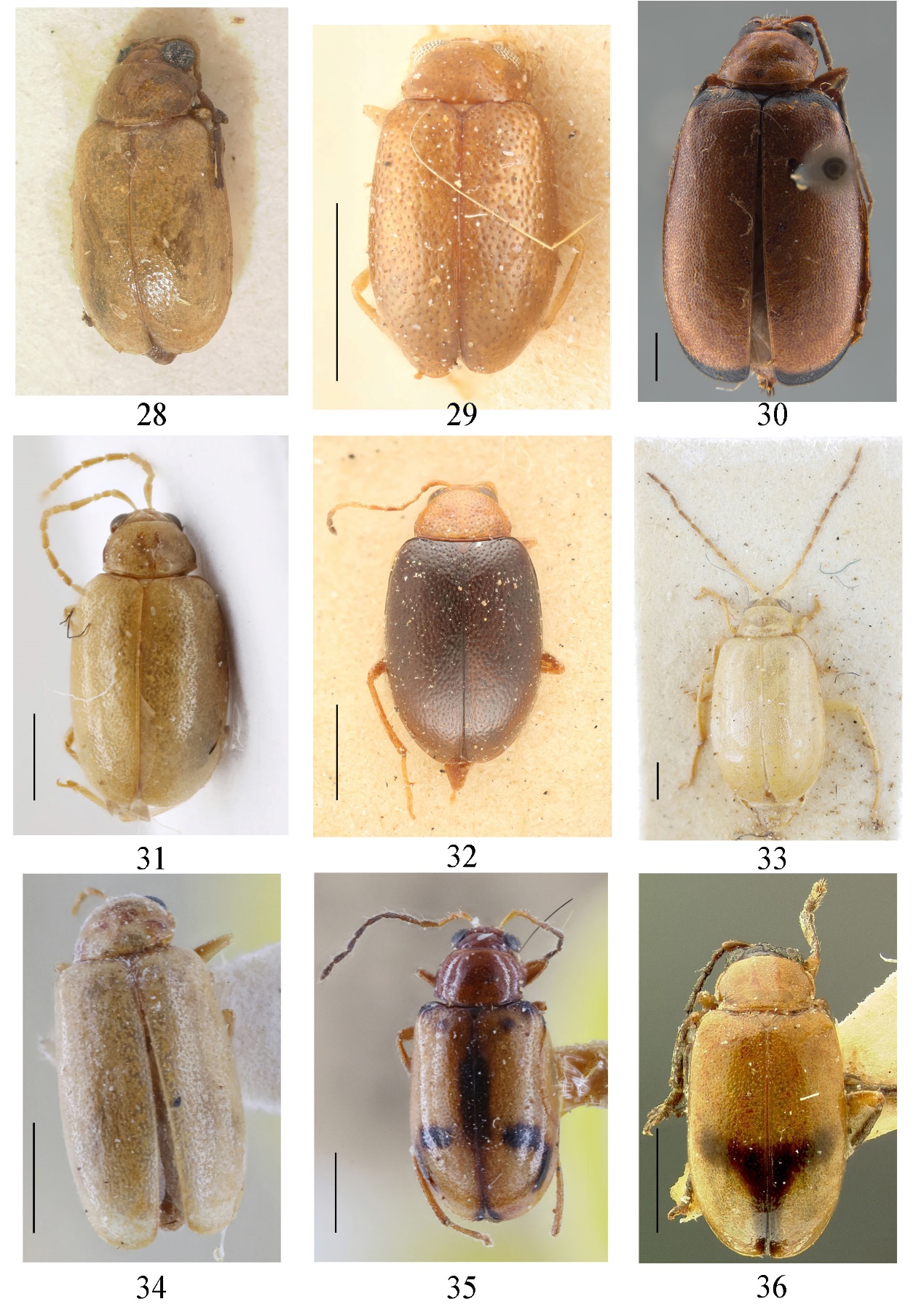
**

**Figures S28–36.** Habitus. **28** *M. minor* (Syntypus, NSNM) **29** *M. minutissima* (Holotype, IZAS) **30** *M. occifluvis* (Holotype, CAS) **31** *M. ongi* (identified species) **32** *M. ovatula* (Holotype, IZAS) **33** *M. pallidula* (Syntype, BMNH) **34** *M. palliparva* (Paratype, BMNH) **35** *M. parenthetica* (Paratype, BMNH) **36** *M. postfasciata* (Holotype, MCZ). Scale bar: 1 mm.

**
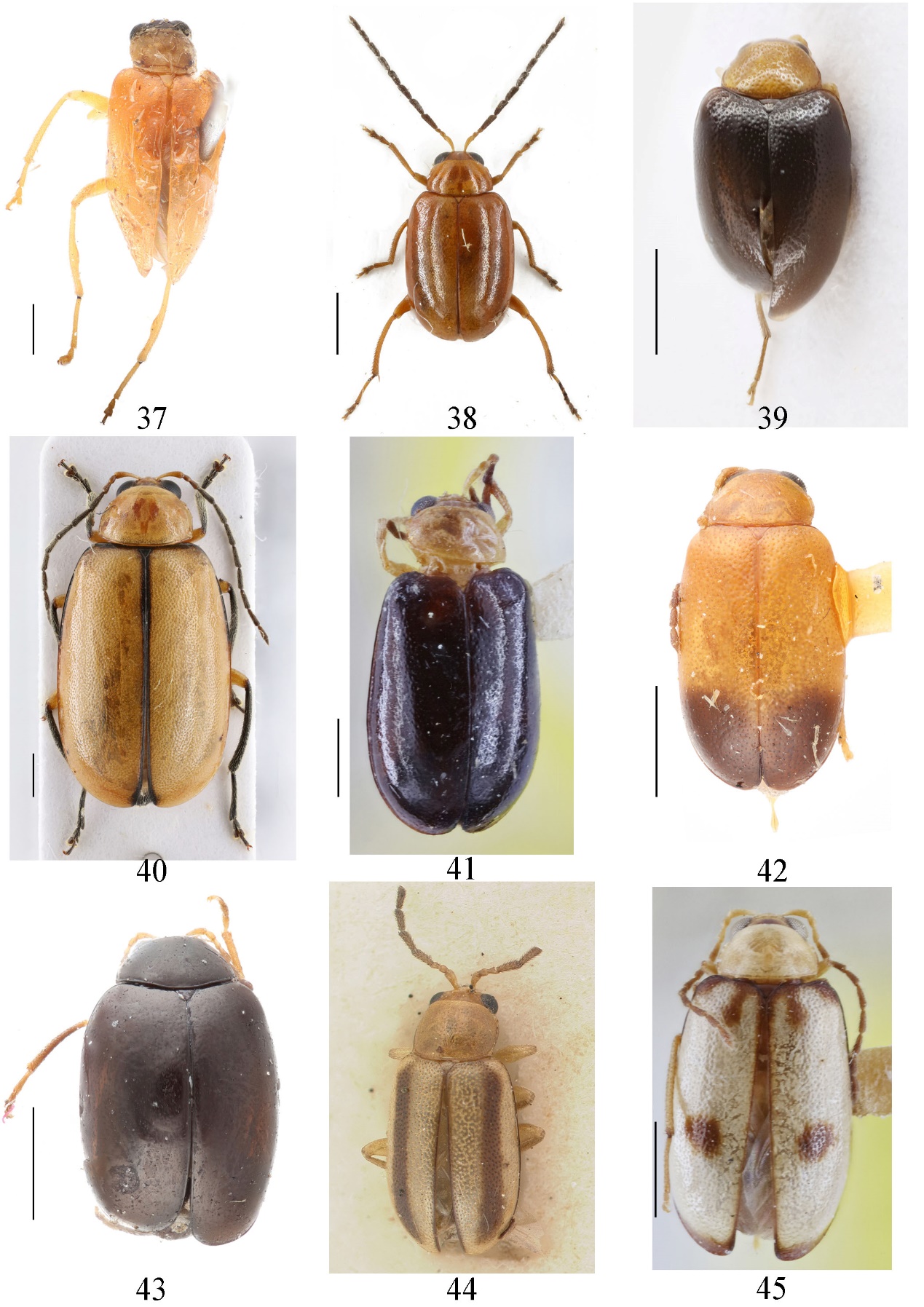
**

**Figures S37–45.** Habitus. **37** *M. quadricavata* (Holotype, IZAS) **38** *M. rufofulva* (identified species) **39** *M. sasajii* (identified species) **40** *M. sauteri* (identified species) **41** *M. schereri* (Paratype, BMNH) **42** *M. selmani* (Paratype, IZAS) **43** *M. semenovi* (identified species) **44** *M. sexlineata* (Syntypus, NSNM) **45** *M. shaowuensis* (Paratype, BMNH). Scale bar: 1 mm.

**
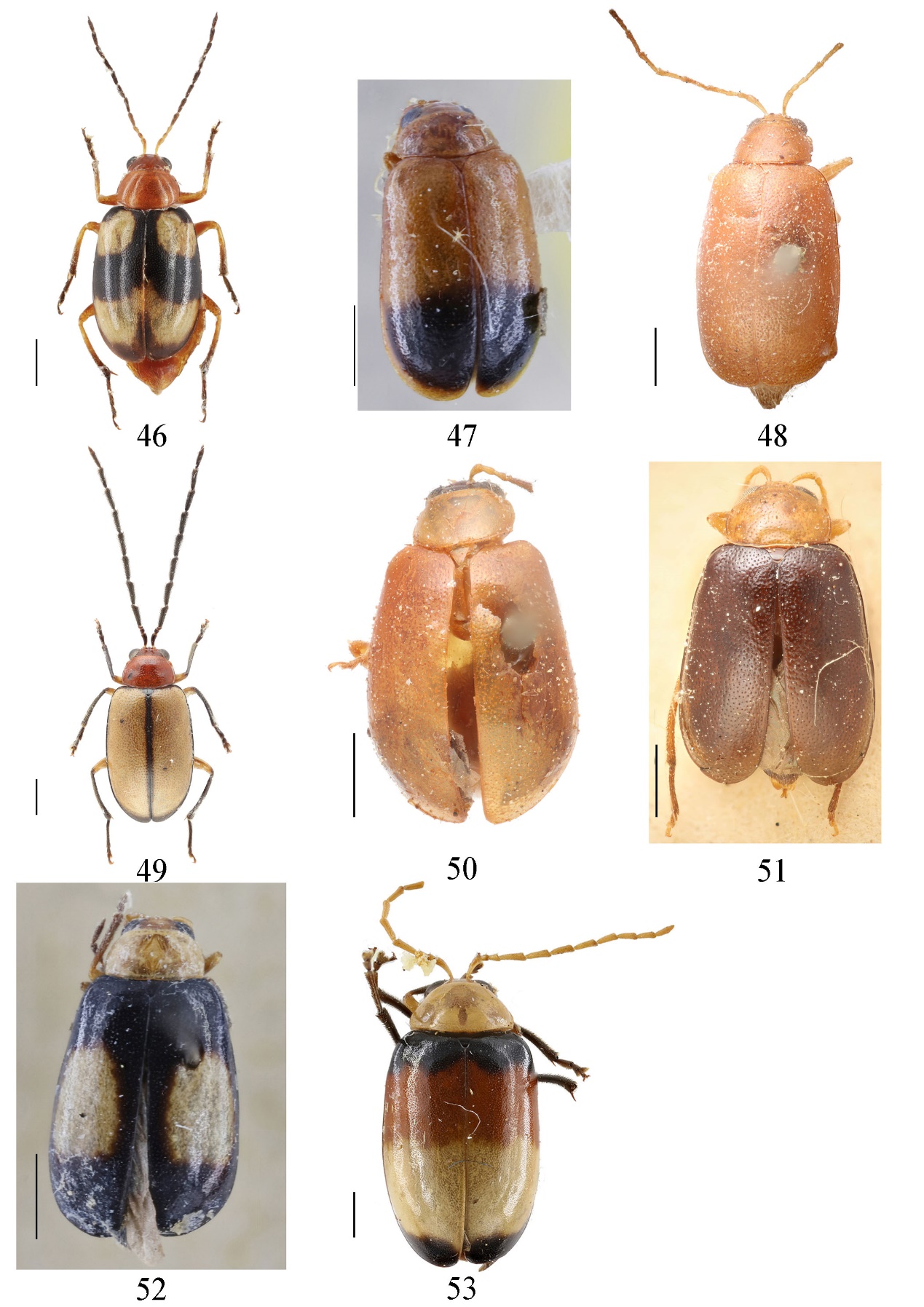
**

**Figures S46–53.** Habitus. **46** *M. signata* (identified species) **47** *M. subapicalis* (Paratype, BMNH) **48** *M. subrubra* (Holotype, IZAS) **49** *M. wilcoxi* (identified species) **50** *M. xanthodera* (Holotype, IZAS) **51** *M. yaosanica* (Holotype, IZAS) **52** *M. yunnanica* (Paratype, BMNH) **53** *M. zonalis* (identified species). Scale bar: 1 mm.

**Notes.**

Some photographs are from museum websites or were taken by the colleagues:

NSNM (from http://twinsecttype.nmns.edu.tw/?tdsourcetag=s_pcqq_aiomsg): *M. horni* (Syntypus, Fig. A14), *M. longitarsoides* (Syntypus, Fig. A23), *M. minor* (Syntypus, Fig. A28), *M. sexlineata* (Syntypus, Fig. A44).

CAS (taken by Christopher Grinter): *M. arundinariae* (Holotype, Fig. A3), *M. brittoni* (Holotype, Fig. A4), *M. lunata* (Holotype, Fig. A25), *M. meridionalis* (Holotype, Fig. A27), *M. occifluvis* (Holotype, Fig. A30).

MCZ (from http://140.247.96.247/mcz/recordlist.php?-skip=16315&-max=5): *M. leechi* (Holotype, Fig. A19), *M. postfasciata* (Holotype, Fig. A36).

USNM (from <https://collections.nmnh.si.edu/search/ento/>): *M. liui* (Holotype, Fig. A21).
